# Reduced Acrolein Detoxification in *akr1a1a* Zebrafish Mutants Causes Impaired Insulin Receptor Signaling and Microvascular Alterations

**DOI:** 10.1002/advs.202101281

**Published:** 2021-07-18

**Authors:** Haozhe Qi, Felix Schmöhl, Xiaogang Li, Xin Qian, Christoph T. Tabler, Katrin Bennewitz, Carsten Sticht, Jakob Morgenstern, Thomas Fleming, Nadine Volk, Ingrid Hausser, Elena Heidenreich, Rüdiger Hell, Peter Paul Nawroth, Jens Kroll

**Affiliations:** ^1^ Department of Vascular Biology and Tumor Angiogenesis European Center for Angioscience (ECAS) Medical Faculty Mannheim Heidelberg University Mannheim 68167 Germany; ^2^ Department of Vascular Surgery Renji Hospital School of Medicine Shanghai Jiaotong University Shanghai 200127 China; ^3^ NGS Core Facility Medical Faculty Mannheim Heidelberg University Mannheim 68167 Germany; ^4^ Department of Internal Medicine I and Clinical Chemistry Heidelberg University Hospital Heidelberg 69120 Germany; ^5^ German Center for Diabetes Research (DZD) Neuherberg 85764 Germany; ^6^ Tissue Bank of the National Center for Tumor Diseases (NCT) Heidelberg Heidelberg University Heidelberg 69120 Germany; ^7^ Institute of Pathology IPH EM Lab Heidelberg University Hospital Heidelberg 69120 Germany; ^8^ Metabolomics Core Technology Platform Centre for Organismal Studies Heidelberg University Heidelberg 69120 Germany; ^9^ Joint Heidelberg‐IDC Translational Diabetes Program Helmholtz‐Zentrum Neuherberg 85764 Germany

**Keywords:** Acrolein (ACR), diabetes, impaired glucose homeostasis, organ complications, zebrafish

## Abstract

Increased acrolein (ACR), a toxic metabolite derived from energy consumption, is associated with diabetes and its complications. However, the molecular mechanisms are mostly unknown, and a suitable animal model with internal increased ACR does not exist for in vivo studying so far. Several enzyme systems are responsible for acrolein detoxification, such as Aldehyde Dehydrogenase (ALDH), Aldo‐Keto Reductase (AKR), and Glutathione S‐Transferase (GST). To evaluate the function of ACR in glucose homeostasis and diabetes, *akr1a1a^−/−^* zebrafish mutants are generated using CRISPR/Cas9 technology. Accumulated endogenous acrolein is confirmed in *akr1a1a^−/−^* larvae and livers of adults. Moreover, a series of experiments are performed regarding organic alterations, the glucose homeostasis, transcriptome, and metabolomics in *Tg(fli1:EGFP*) zebrafish. *Akr1a1a^−/−^* larvae display impaired glucose homeostasis and angiogenic retina hyaloid vasculature, which are caused by reduced acrolein detoxification ability and increased internal ACR concentration. The effects of acrolein on hyaloid vasculature can be reversed by acrolein‐scavenger l‐carnosine treatment. In adult *akr1a1a^−/−^* mutants, impaired glucose tolerance accompanied by angiogenic retina vessels and glomerular basement membrane thickening, consistent with an early pathological appearance in diabetic retinopathy and nephropathy, are observed. Thus, the data strongly suggest impaired ACR detoxification and elevated ACR concentration as biomarkers and inducers for diabetes and diabetic complications.

## Graphical abstract

Acrolein (ACR) plays a critical role in diabetes processing. This work generates an *akr1a1a* knockout zebrafish model and shows that the loss of Akr1a1a leads to an impaired detoxification and accumulation of ACR in vivo, illustrating that internally increased ACR causes altered glucose homeostasis, which finally induces pathological retinal angiogenesis and thickening of the glomerular basement membrane (GBM).

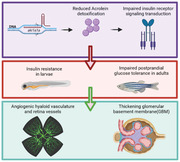

## 1 Introduction

Diabetes is a worldwide disease and is characterized by a high blood glucose level over a prolonged period of time. More than 463 million people have been diagnosed with diabetes so far, and the number will be rising to 700 million in 2045 as estimated.^[^ ^1^ ^]^ Without appropriate treatment in time, diabetes can cause severe microvascular complications in the eye and kidney, namely diabetic retinopathy and nephropathy, which become the leading cause of vision loss and renal failure in working‐age people.^[^ ^2^ ^]^ Hence, early diagnosis and intervention of diabetes are essential and urgently needed for improving long‐term prognosis.

Increasing evidence indicates that reactive carbonyl species (RCS) are positively correlated with diabetes and insulin resistance.^[^ ^3^, ^4^, ^5^, ^6^, ^7^, ^8^ ^]^ Among them, methylglyoxal (MG) is well studied and regarded as a main toxic factor.^[^ ^9^, ^10^ ^]^ In addition to MG, acrolein (ACR) also has drawn extreme attention due to its reactive capacity in past decades.^[^ ^11^, ^12^ ^]^ ACR, originated from myeloperoxidase‐mediated degradation of threonine, amine oxidase‐mediated degradation of spermine and spermidine, and lipid peroxidation of polyunsaturated fatty acids (PUFAs) in vivo, was considered as a toxic intermediate which plays certain roles in Alzheimer's diseases, neuropathic pain, spinal cord injury, cardiovascular disease, and diabetes.^[^ ^13^, ^14^, ^15^, ^16^, ^17^ ^]^

Preliminary studies have identified that ACR‐lysine adduct (FDP‐lysine) elevates in type 1 and type 2 diabetic patients.^[^ ^18^, ^19^ ^]^ ACR‐relevant metabolites, such as *N*‐acetyl‐S‐(3‐hydroxypropyl)‐l‐cysteine (3‐HPMA) and *N*‐acetyl‐S‐(carboxyethyl)‐l‐cysteine (CEMA), were positively associated with diabetes as well as insulin resistance by dose‐dependent response.^[^ ^20^ ^]^ Additionally, FDP‐lysine level has a strong connection with diabetic nephropathy and retinopathy.^[^ ^21^, ^22^ ^]^ Although studies have revealed an apparent connection between ACR and diabetic complications, whether ACR contributes to the onset of diabetes and organ damage remained unknown. Therefore, a suitable animal model with increased ACR is necessary for further in vivo exploration.

Typically, the steady‐state concentration of ACR is maintained in a certain range. However, changes occurring in ACR production or detoxification procedure by its corresponding enzymes, such as Aldo‐Keto Reductase (AKR), Aldehyde Dehydrogenase (ALDH), and Glutathione S‐Transferase (GST), may lead to its accumulation and damaging effects on proteins, nucleic acids, and lipids afterward.^[^ ^13^, ^23^, ^24^ ^]^

The first discovered AKR, the Aldehyde Reductase Akr1a1, is a cytosolic, NADPH‐dependent, monomeric oxidoreductase, which has two separate homologs in zebrafish, namely, Akr1a1a and Akr1a1b.^[^ ^25^, ^26^ ^]^ Akr1a1 possesses a broad substrate spectrum, and it prefers carboxyl‐group containing negatively charged substrates.^[^ ^27^, ^28^ ^]^ In addition to detoxifying common RCS, such as 3‐deoxyglucosone (3DG), glyoxal, and MG, Akr1a1 also has a high affinity to ACR.^[^ ^25^ ^]^ Kurahashi et al. reported that the detoxification of toxic aldehydes, for example, ACR, is a principal function for Akr1a1, and overexpression of Akr1a1 alleviates the *Tg MEF*’s (transgenic Mouse Embryonic Fibroblasts) sensitivity to ACR.^[^ ^29^ ^]^ In a previous study, Akr1a1b was identified as a gluconeogenesis regulator via adjusting S‐nitrosyaltion in zebrafish.^[^ ^26^ ^]^ However, whether Akr1a1a functions closely to Akr1a1b or completely separate and whether a permanent loss of Akr1a1a causes accumulated internal ACR and results in diabetes and relevant complications in the end require further elucidation.

Therefore, the study aimed to establish an animal model with increased internal levels of ACR and clarify the subsequent effects of ACR on glucose metabolism and organic alterations in zebrafish. Our data indicate impaired ACR detoxification and accumulated ACR as inducers for insulin resistance and resulted in the pathological progression of diabetic retinopathy and nephropathy.

## 2 Results

### 2.1 Expression of *akr1a1a* in Zebrafish and Generation of *akr1a1a* Knockout Zebrafish

**Figure 1:**
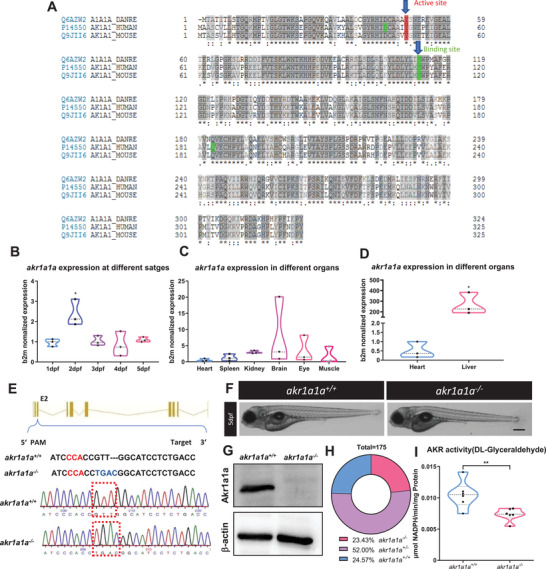
Sequence alignment of Akr1a1a across different species and generation of Akr1a1a knockout zebrafish by using CRISPR‐Cas9 technology. A) The amino acid alignment showed a high similarity between the different species on the active site (red frame) and binding site (green); first line: zebrafish AKR1a1a; second line: human AKR1a1; third line: mouse AKR1a1. B) *akr1a1a* mRNA expression in wild‐type zebrafish larvae showed a significant upregulation at 2 dpf. C,D) *akr1a1a* mRNA expressed mostly in liver of wild‐type adult zebrafish (heart as reference organ). Expression of genes was determined by RT‐qPCR and normalized to *b2m*. Larval stage: *n* = 3 clutches with 30 larvae, adult organs: *n* = 3 with one organ per sample. E) Akr1a1a‐CRISPR‐target site was designed in exon 2 of the *akr1a1a* gene and CRISPR/Cas9‐induced deletion‐insertion of four nucleotides was selected for further *akr1a1a* mutant line generation and maintenance. Genotype was analyzed via sequencing chromatograms of PCR‐amplified *akr1a1a* region, containing the *akr1a1a* target site. Chromatogram shows *akr1a1a* wild type and deletion‐insertion of four nucleotides homozygous sequencing results. F) Microscopic images showed unaltered morphology of *akr1a1a^−/−^* larvae in comparison with *akr1a1a^+/+^* larvae at 5dpf. Black scale bar: 300 µm. G) Western blot for Akr1a1a expression in adult liver showed the loss of Akr1a1a protein in mutants. b‐actin served as loading control. *n* = 3, each lane represents one liver sample from according adult fish. H) Adult fish number among different genotypes was in line with *the Mendelian Inheritance* in the first generation of F2: *akr1a1a^+/+^* = 43, *akr1a1a^+/‐^* = 91, *aldh3a1^−/−^* = 41. I) *akr1a1a^−/−^* zebrafish showed decreased enzyme activity (DL‐Glyceraldehyde served as substrate) measured by spectrophotometric analysis in zebrafish lysates at 96 hpf; *n* = 6–11 clutches, each clutch contains 50 larvae. For statistical analysis one‐way ANOVA followed by Tukey's multiple comparison test and Student's *t*‐test was applied, *p < 0.05. **p < 0.01. RT‐qPCR, real‐time quantitative polymerase chain reaction; dpf, day post fertilization; b2m, *β*2 microglobulin. PAM, protospacer‐adjacent motif.

In zebrafish, two homologs for Akr1a1, including Akr1a1a and Akr1a1b, exist. The alignment comparing the amino acid sequence of Akr1a1 and Akr1a1a in humans, mice, and zebrafish showed that zebrafish Akr1a1a shares a 60% and 58% similarity with Akr1a1 in humans and mice, respectively. Meanwhile, Akr1a1a and Akr1a1 possess the same active site and binding site among three different species, suggesting Akr1a1a as a potential candidate to study ACR detoxification in zebrafish (**Figure** **1**A).

*Akr1a1a* mRNA expression was determined by RT‐qPCR in larvae and adult organs of zebrafish. The results showed ubiquitous *akr1a1a* expression throughout embryonic and larval stages with being highest expressed at 2dpf (Figure 1B). Besides, *akr1a1a* expression was mostly observed in the liver, more than two hundred‐fold than in the reference organ (heart), and to a less extent in the brain (eightfold), kidney (threefold), and eye (threefold) (Figure 1C,D). Altogether, these data suggest that Akr1a1a distributes widely in the early developmental stages of zebrafish larvae and adult organs, and it may play an essential role in embryonic development and in physiological liver function.

In order to explore the function of Akr1a1a in zebrafish and on ACR metabolism, an *akr1a1a^−/−^* mutant line was generated by CRISPR/Cas9 technology as the first step.^[^ ^30^ ^]^ Briefly, gRNA was designed targeting exon 2 of *akr1a1a*, and a deletion‐insertion of four nucleotides in the *Tg(fli1: EGFP)* reporter line was identified and utilized for further studies (Figure 1E). The general morphology of larvae at 5dpf did not show any noticeable alterations in mutants compared to the wild types (Figure 1F). To evaluate whether the *akr1a1a* mutations caused a non‐functional Akr1a1a protein after translation, a Western blot was performed and showed an ultimate loss of the Akr1a1a protein in mutants (Figure 1G). The percentage of *akr1a1a^−/−^* zebrafish growing into adulthood is about 23.43%, while 24.57% to wild type and 52% to heterozygous zebrafish, which is consistent with Mendel's law of inheritance suggesting permanent loss of *akr1a1a* does not affect the survival of zebrafish (Figure 1H). Besides, AKR activity was measured by using DL‐Glyceraldehyde as substrate, and it showed significant decline in *akr1a1a^−/−^* larvae (Figure 1I). All the above data have proven the successful generation of *akr1a1a* knockout mutants.

### 2.2 Alteration of the Retinal Vasculature and Glomerular Basement Membrane in *akr1a1a^−/−^* Mutants

**Figure 2:**
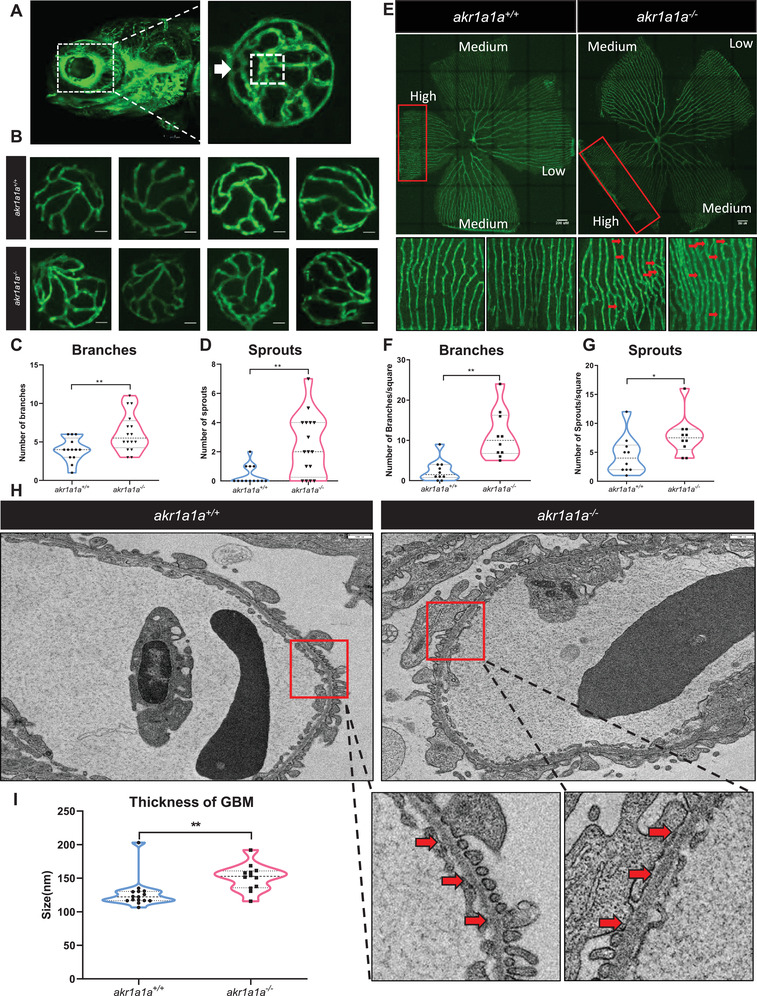
Retinal vasculature and renal alterations in *akr1a1a^−/−^ Tg(fli1:EGFP)* zebrafish larvae and adults. A) Representative confocal images of the hyaloid vasculature in zebrafish larvae. White box: central hyaloid/optic artery. White arrow: circumferential inner annular hyaloid vessel. B) Representative confocal images of the hyaloid vasculature in *akr1a1a^+/+^* and *akr1a1a^−/−^* larvae show vascular alterations in the mutants. White scale bar = 20 µm. C,D) Quantification of increased branches and sprout formation in the hyaloid vasculature in *akr1a1a^−/−^* larvae, *n* = 10–16 per group. E) Representative confocal images of retinal vasculature in *akr1a1a^+/+^* and *akr1a1a^−/−^* adults. White scale bar = 200 µm. F,G) Quantification of increased branches and sprout formation in the adult retinal vasculature in *akr1a1a^−/−^* zebrafish, *n* = 13–16 per group. H,I) Representative electron microscopy images and quantification showed thicker GBM in adult *akr1a1a^−/−^* kidneys. Red arrows: GBM. White scale bar, 500 nm. For statistical analysis Student's *t*‐test was applied. *p < 0.05, **p < 0.01.

Diabetic retinopathy and diabetic nephropathy are common microvascular alterations and zebrafish has been established as a valuable animal model to study alterations in the eyes and kidneys.^[^ ^31^, ^32^ ^]^ The hyaloid vasculature was analyzed first by using the *Tg(fli1: EGFP)* reporter line.^[^ ^33^ ^]^ Zebrafish larvae were collected at 5 dpf, and images were captured with a confocal microscope. Increasing numbers of branches and sprouts were identified in the hyaloid vasculature of *akr1a1a^−/−^* larvae compared to *akr1a1a^+/+^* larvae at 5 dpf (**Figure** **2**A–D). Meanwhile, as same as in larvae, adult *akr1a1a^−/−^* zebrafish also displayed more branches and sprouts in retinal vessels in contrast to *akr1a1a^+/+^* adults (Figure 2E–G). Besides, although no apparent alterations in kidneys have been found with PAS staining under the light microscope (Figure S1A,B, Supporting Information), a thickening of the glomerular basement membrane (GBM) was observed using electron microscopy in *akr1a1a^−/−^* adults (Figure 2H,I). Taken together, these results revealed that the loss of Akr1a1a leads to alterations of the hyaloid vasculature in zebrafish larvae, which persists into adulthood, besides, the loss of Akr1a1a resulted in a thickening of the GBM in adults. Thus, *akr1a1a* mutants show incipient hallmarks of diabetic retinopathy and diabetic nephropathy.

### 2.3 Insulin Receptor Signaling Pathway Was Down‐Regulated in *akr1a1a^−/−^* Larvae

**Figure 3:**
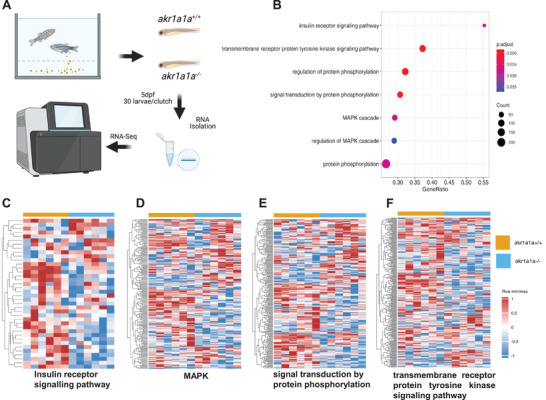
Down‐regulated insulin receptor signaling pathway and downstream pathways in *akr1a1a^−/−^* zebrafish larvae. A) Experimental design for larval RNA‐Seq. 30 larvae per clutch, 6 clutches of *akr1a1a^+/+^* and *akr1a1a^−/−^* zebrafish larvae at 5dpf were applied for RNA isolation. B) Bubble plot showed the significantly down‐regulated biological pathways in *akr1a1a^−/−^* zebrafish larvae at 5dpf via KEGG and GOBP analysis. Heatmaps showed relative mRNA expression in C) insulin receptor signaling pathway, D) MAPK, E) signal transduction by protein phosphorylation, and F) transmembrane receptor protein tyrosine kinase signaling pathway was down‐regulated significantly in *akr1a1a^−/−^* zebrafish larvae. Higher and lower expression is displayed in red and blue, respectively. GSEA, gene set enrichment analysis. dpf, day post fertilization. KEGG, Kyoto encyclopedia of gene and genomes. GOBP, Gene ontology biological processes.

To investigate the potential mechanisms behind the alterations in *akr1a1a^−/−^* mutants, gene‐expression patterns were analyzed by genome RNA‐Seq among *akr1a1a^+/+^* and *akr1a1a^−/−^* larvae at 5dpf (**Figure** **3**A). Principal component analysis (PCA) exhibited components of each sample by which showed that *akr1a1a^+/+^* and *akr1a1a^−/−^* plots are totally separated in the PC1 axis (Figure S2A, Supporting Information). Quality control showed comparable properties between *akr1a1a* mutants and wild types (Figure S2B, Supporting Information). Then, gene set enrichment analysis (GSEA) was performed to better understand altered physiological signaling pathways reflected by the loss of Akr1a1a. Among a series of altered biological pathways, the insulin receptor and downstream signaling pathways, including but not limited to MAPK, signal transduction by protein phosphorylation and transmembrane receptor protein tyrosine kinase signaling pathway, were significantly down‐regulated in *akr1a1a* mutants (Figure 3B–F), suggesting the loss of Akr1a1a induces an impaired insulin signaling transduction in zebrafish larvae at 5dpf. Additionally, metabolomics assay was also performed in wild type and homozygous larvae and livers. Among amino acids, thiols, adenosines and fatty acids, lysine, putrescin and C20:3n6 were significantly increased in mutant larvae. C18:3n6, C20:3n6 were significantly increased while cholesterol decreased significantly in mutants’ liver which implies the impaired insulin signaling transduction in *akr1a1a^−/−^* larvae give rises to alterations in protein and fatty acids synthesis and metabolism procedures (Figures S3,S4, Supporting Information).

### 2.4 *Akr1a1a^−/−^* Mutants Displayed Impaired Glucose Homeostasis and Accumulated Internal ACR

**Figure 4:**
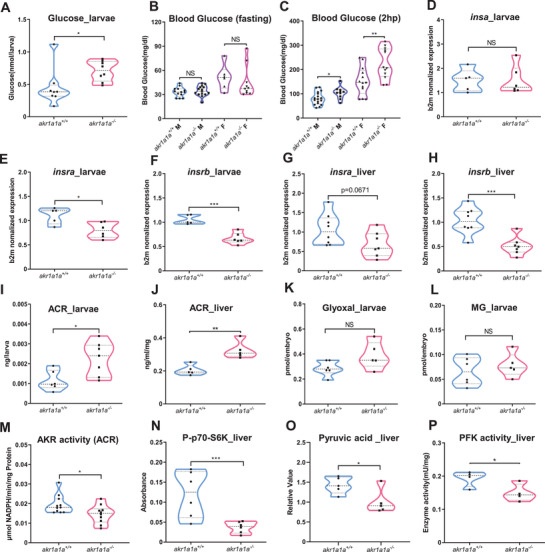
Altered glucose and insulin related gene expression in *akr1a1a^−/−^* zebrafish. A) *Akr1a1a^−/−^* larvae showed significantly increased whole‐body glucose at 5dpf. *n* = 8 clutches with 20 larvae. B) Blood glucose of *akr1a1a^−/−^ a*dults did not show any alteration in fasting state in both male and female. *n* = 6–15. C) In the postprandial state, *akr1a1a^−/−^* adults displayed higher blood glucose than *akr1a1a^+/+^* adults in both male and female. *n* = 10–17. D) *insa* gene expression level showed no alteration in *akr1a1a^−/−^* larvae compared with *akr1a1a^+/+^* larvae at 5 dpf; *n* = 5–6 clutches with 30 larvae. E,F) Both *insra* and *insrb* mRNA expression were down‐regulated in *akr1a1a^−/−^* larvae at 5dpf. *n* = 5–6 clutches with 30 larvae. G,H) *insra* mRNA expression showed a declining trend while *insrb* mRNA expression was down‐regulated significantly in *akr1a1a^−/−^* adult livers. *n* = 7–8. I) ACR was significantly increased in *akr1a1a^−/−^* larvae. *n* = 6–7 clutches with 50 larvae. J) ACR accumulated significantly in the liver of *akr1a1a^−/−^* adults, *n* = 5. K,L) Glyoxal and MG were unaltered in *akr1a1a^−/−^* larvae. *n* = 5–6 clutches with 50 larvae. M) AKR activity (ACR served as substrate) was significantly reduced in *akr1a1a^−/−^* larvae. *n* = 5–6 clutches with 50 larvae. N) Phosphorylated p70‐S6K(P‐p70‐S6K) was significantly decreased in the liver of *akr1a1a^−/−^* adults, *n* = 6. O) Pyruvic acid was significantly decreased in the liver of *akr1a1a^−/−^* adults, *n* = 5. P) PFK activity reduced significantly in the liver of *akr1a1a^−/−^* adults. For statistical analysis Student's *t*‐test was applied. *p < 0.05. **p < 0.01. ***p < 0.001. NS, not significant.

Although impaired insulin signaling transduction has been verified in *akr1a1a^−/−^* mutants, whether glucose homeostasis alters afterward is still unknown. In order to address this question, body glucose and blood glucose measurements were performed in larvae and adults, respectively. Interestingly, *akr1a1a^−/−^* larvae exhibited 50% incremental glucose level in contrast to *akr1a1a^+/+^* larvae at 5dpf (**Figure** **4**A). Additionally, *akr1a1a^−/−^* adults displayed postprandial hyperglycemia in both male and female, while overnight‐fasting blood glucose kept unaltered (Figure 4B,C) proving that permanent loss of Akr1a1a can induce impaired glucose homeostasis in both zebrafish larvae and adults.

To explore the potential leading cause for impaired glucose homeostasis in *akr1a1a^−/−^* mutants, *preproinsulin* (*insa*), and *insulin receptor* (*insra, insrb*) mRNA expression levels were analyzed by RT‐qPCR. Since *akr1a1a* has been seen expressed mostly in liver (Figure 1D), larvae and liver were chosen as target organs. Meanwhile, potential substrates for Akr1a1a including ACR, glyoxal and methylglyoxal were measured accordingly. Intriguingly, *insa*, the main insulin‐encoding gene involved in glucose homeostasis regulation in zebrafish, was unaltered in *akr1a1a^−/−^* larvae, but *insra* and *insrb* mRNA reduced significantly in *akr1a1a^−/−^* larvae at 5 dpf (Figure 4D–F). Moreover, reduced *insrb* mRNA expression, and decreased *insra* mRNA expression tendency was also found in adult *akr1a1a^−/−^* livers, suggesting reduced *insra/insrb* expression as the cause for impaired glucose homeostasis and altered insulin signaling transduction in *akr1a1a* mutants (Figure 4G,H). Importantly, accumulated protein‐bound ACR in larvae and livers, but not glyoxal and methylglyoxal, was identified in *akr1a1a^−/−^* mutants (Figure 4I–L). In addition, decreased AKR activity by utilizing ACR as substrate was observed in *akr1a1a^−/−^* larvae proving Akr1a1a as a main metabolizing enzyme for ACR in zebrafish (Figure 4M). Furthermore, the decline of phosphorylated p70S6K and pyruvic acid confirmed impaired glucose homeostasis and hinted the existence of insulin resistance in *akr1a1a^−/−^* livers (Figure 4N,O). However, expression of important glycolytic enzymes, such as *phosphofructokinase* (*pfk*) and *hexokinase* (*hk1*) were not altered while *pyruvate kinase* (*pk*) was increased in *akr1a1a^−/−^* livers (Figure S5A, Supporting Information). In addition to the expression analysis, enzyme activity determination was also performed for phosphofructokinase (PFK), pyruvate kinase (PK), and glucokinase (GK). The results indicated that PFK activity but not PK and HK, decreased significantly in livers of mutants suggesting glycolysis procedure is also partially influenced after Akr1a1a knockout (Figure 4P; Figure S5B,C, Supporting Information).

Last, different from previous studies regarding Akr1a1b,^[^ ^26^ ^]^ the entirely loss of Akr1a1a did not up‐regulate S‐nitrosylated proteins (Figure S6, Supporting Information). At the meantime, *akr1a1b^−/−^* mutants displayed unaltered *insra/insrb* mRNA expression, internal ACR level and normal hyaloid vasculature (Figures S7,S8, Supporting Information). All these evidence suggest that zebrafish Akr1a1a is not the functional homologue to zebrafish Akr1a1b and mouse Akr1a1 in regualting S‐Nitrosylation.^[^ ^34^ ^]^ Meanwhile, Glo1 and ALDH enzyme activities were also determined since these two enzyme systems exert similar functions as the AKR enzyme system in detoxifying RCS metabolites and AGEs precursors. However, Glo1 and ALDH enzyme activity kept unchanged in *akr1a1a^−/−^* larvae compared to *akr1a1a^+/+^* larvae at 4 dpf suggesting Glo1 and ALDH enzyme systems are not able to detoxify accumulated ACR in zebrafish (Figure S9A,B, Supporting Information).

### 2.5 *Insra/insrb* Expression Silencing Induced an Angiogenic Hyaloid Vasculature and Impaired Glucose Homeostasis in Zebrafish Larvae

**Figure 5:**
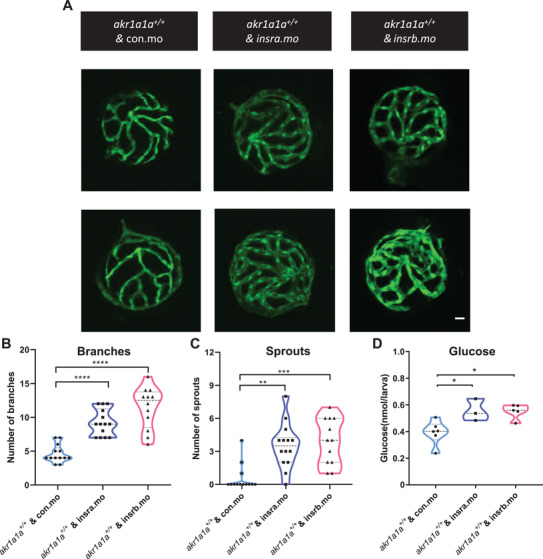
*insra/insrb expression* silencing induced vascular alterations and caused higher glucose level at 5 dpf. A) Representative confocal images of hyaloid vasculature. White scale bar: 20 µm. B,C) Quantification of hyaloid vasculature showed significant increasing numbers of branches and sprouts in larvae upon *insra/insrb* silencing at 5 dpf. *n* = 12–14. D) Whole‐body glucose measurement showed higher glucose level in *akr1a1a1^+/+^* larvae upon *insra/insrb* silencing at 5 dpf. *n* = 3–6 clutches with 20 larvae. For statistical analysis one‐way ANOVA followed by Tukey's multiple comparisons test was applied. *p < 0.05, **p < 0.01, ***p < 0.001, ****p < 0.0001.

In order to address whether down‐regulated *insra/insrb* expression resulted in glucose homeostasis impairment and were responsible for the angiogenic phenotypes appearing in *akr1a1a^−/−^* zebrafish, *insra* and *insrb* morpholinos (SB‐*insra*‐MO, SB‐*insrb*‐MO) were designed and used as tools to transiently and partially silence *insra* and *insrb* expression by antisense approach. SB‐*insra*‐MO and SB‐*insrb*‐MO target exon3‐intron3 and exon7‐intron7 junctions of *insra/insrb*, respectively (Figure S10A, Supporting Information). Two nanograms of morpholinos were injected into the one‐cell stage of wild‐type zebrafish embryos. The efficiency of morpholinos was validated by RT‐PCR, which showed decreased wild type *insra/insrb* and the elevated expression of morphant *insra/insrb* upon SB‐*insra*‐MO and SB‐*insrb*‐MO injection (Figure S10B, Supporting Information). Hyaloid vasculature was then analyzed after *insra/insrb* morpholino injection. Interestingly, similar to *akr1a1a^−/−^* larvae, either *insra* or *insrb* silencing could lead to alterations in wild type larvae's hyaloid vasculature (**Figure** **5**A–C). Besides, whole‐body glucose increased significantly in both *insra/insrb* silencing groups at 5 dpf (Figure 5D). In addition, AKR enzyme activity was determined in *insra/insrb* morphants. It turned out that by taking common substrate, DL‐Glyceraldehyde, AKR activity did not show a detectable alteration while it displayed a decline in both morphants by using ACR as substrate (Figure S11, Supporting Information). To sum up, these data indicated the downregulation of *insra* and *insrb* resulting from permanent loss of Akr1a1a induced imbalanced glucose homeostasis and alterations in the hyaloid vasculature. Besides, partial loss of *insra* and *insrb* gene expression may lead to impaired ACR detoxifying capability of AKR enzyme.

### 2.6 ACR Caused Angiogenic Alterations in Hyaloid Vasculature and Impaired Glucose Homeostasis via Reducing *insra/insrb* mRNA Expression

**Figure 6:**
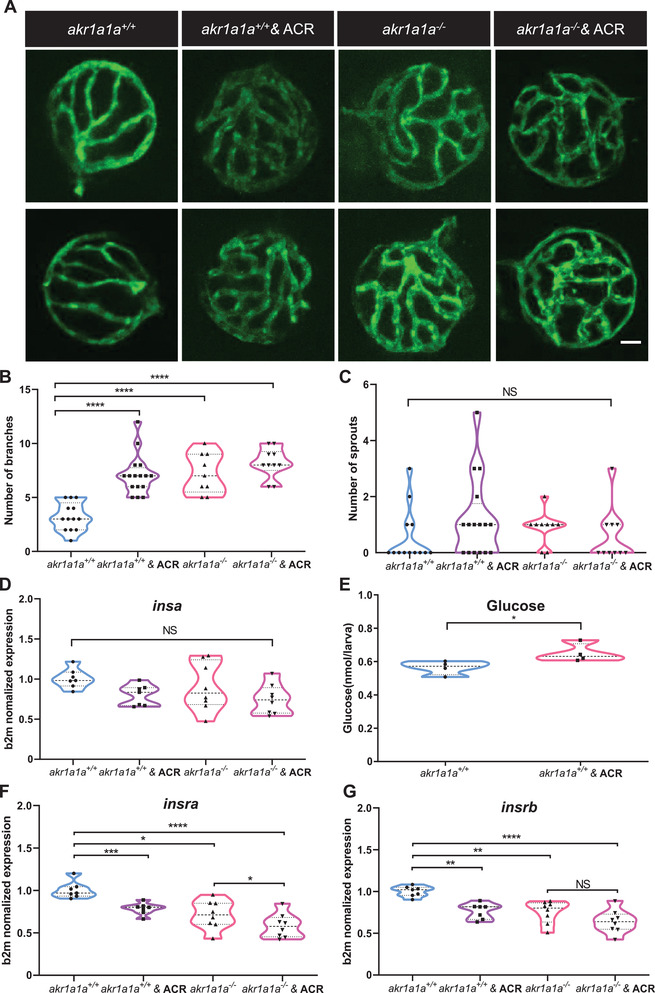
ACR induced alterations on retinal hyaloid vasculature and downregulation of *insra/insrb* mRNA expression at 5dpf. A) Representative confocal images of hyaloid vasculature. White scale bar: 20 µm. B,C) Quantification of hyaloid vasculature showed significant increasing numbers of branches in *akr1a1a1^+/+^* larvae incubated with 10 µm ACR. *n* = 13–16. White scale bar = 20 µm. D) *Insa* mRNA expression was unaltered in *akr1a1a1^+/+^* larvae upon ACR treatment. *n* = 7 clutches with 30 larvae. E) Whole‐body glucose measurement showed higher glucose level in *akr1a1a1^+/+^* larvae upon 10 µm ACR treatment at 5dpf. *n* = 4 clutches with 20 larvae. F,G) Both *insra* and *insrb* showed declined mRNA expression level in *akr1a1a1^+/+^* larvae upon 10 µm ACR treatment. *n* = 7 clutches with 30 larvae. For statistical analysis Student's *t*‐test was applied. *p < 0.05, **p < 0.01, ***p < 0.001, ****p < 0.0001. NS, not significant.

To test the hypothesis if ACR is the missing connection between the declined *insra/insrb* mRNA expression and the loss of Akr1a1a, wild‐type larvae were incubated with exogenous ACR from 1 dpf to 5 dpf. 10 µm was selected as working concentration, since zebrafish exhibited stable survival rate and normal morphology after treatment (Figure S12, Supporting Information). Morphology of hyaloid vasculature was analyzed afterward. Interestingly, more branches were observed in the hyaloid vasculatures after the ACR treatment (**Figure** **6**A–C). Further experiments with ACR intervention showed that treated larvae had normal expression level of *insa* mRNA (Figure 6D), significantly elevated whole‐body glucose (Figure 6E), but less expressed *insra/insrb* mRNA (Figure 6F,G) in contrast to the larvae without treatment, which recapitulates the discoveries in *akr1a1a^−/−^* larvae (Figure 2B).

**Figure 7:**
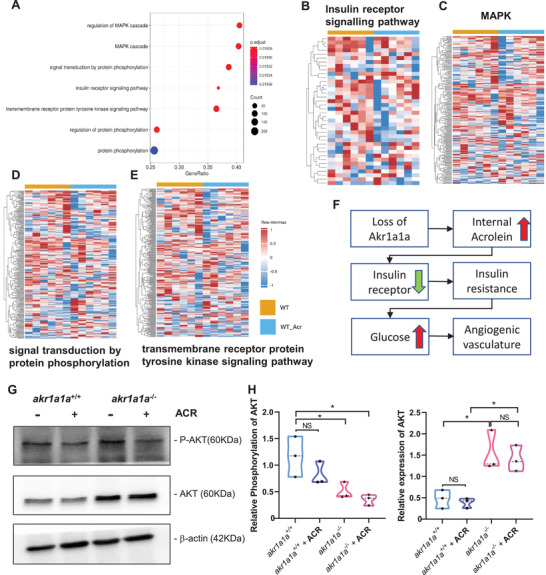
Down‐regulated insulin receptor signaling pathways in *akr1a1a^+/+^* zebrafish larvae upon ACR treatment. A) Bubble plot showed the significantly down‐regulated biological pathways between *akr1a1a^+/+^* and *akr1a1a^+/+^* zebrafish larvae after ACR treatment at 5dpf via KEGG and GOBP analysis. Heatmaps showed relative mRNA expression in B) insulin receptor signaling pathways, C) MAPK, D) signal transduction by protein phosphorylation, and E) transmembrane receptor protein tyrosine kinase signaling pathway was down‐regulated significantly in *akr1a1a^+/+^* zebrafish larvae after ACR treatment. Higher and lower expression is displayed in red and blue, respectively. F) Concise mechanism flow chart showed the consequence of defective ACR detoxification after Akr1a1a loss. G) Representative Western blot shows total AKT and phosphorylated AKT level in different groups. H) Quantification of AKT phosphorylation and total AKT expression in *akr1a1a^+/+^* and *akr1a1a^−/−^* zebrafish larvae treated with ACR. GESA, gene set enrichment analysis. KEGG, Kyoto encyclopedia of gene and genomes. GOBP, gene ontology biological processes. For statistical analysis one‐way ANOVA followed by Tukey's multiple comparisons test was applied. *p < 0.05. NS, not significant.

Additionally, in order to understand how the ACR exerts its functions at the transcriptome level, we performed full genome RNA‐Seq on larvae with or without ACR treatment. Importantly, the insulin receptor and downstream signaling pathways including MAPK, signal transduction by protein phosphorylation and transmembrane receptor protein tyrosine kinase signaling pathway were also down‐regulated (**Figure** **7**A–E), which resemble the earlier findings in *akr1a1a^−/−^* larvae (Figure 3). Furthermore, since AKT/PKB is one of the crucial downstream proteins and is phosphorylated upon insulin‐induced signaling transduction, phosphorylation of AKT/PKB was determined in both *akr1a1a^+/+^ and akr1a1a^−/−^* larvae and in conditions after ACR treatment. The results showed that AKT/PKB expression is increased but phosphorylated AKT/PKB is significantly decreased in *akr1a1a^−/−^* larvae compared to *akr1a1a^+/+^* controls. In contrast to *akr1a1a^+/+^* control larvae, the ACR treated larvae displayed unchanging total AKT/PKB but decreasing tendency for phosphorylated AKT/PKB (Figure 7G,H).

All above data indicated that ACR directly leads to an impaired insulin receptor signaling and disrupts glucose homeostasis. Moreover, accumulated and non‐detoxified internal ACR after the loss of *akr1a1a* is responsible for the impaired glucose homeostasis and altered hyaloid vasculature in *akr1a1a^−/−^* mutants (Figures 2, 6).

### 2.7 Angiogenic Alterations in Hyaloid Vasculature Caused by ACR Can Be Rescued by l‐Carnosine and PK11195

**Figure 8:**
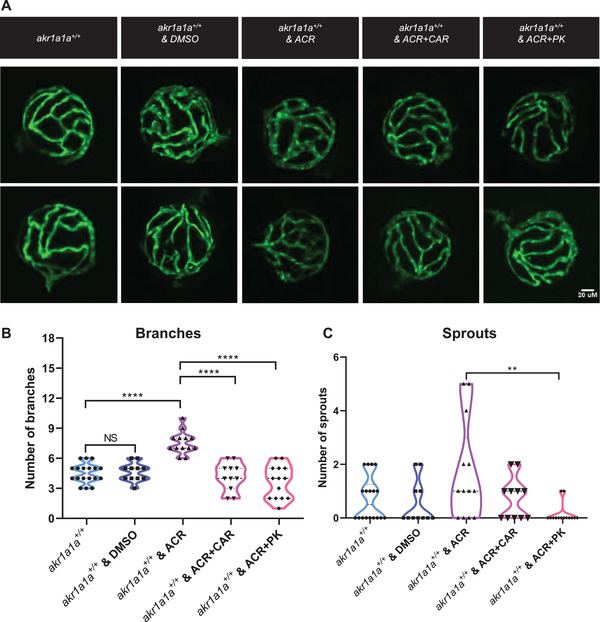
Carnosine and PK11195 can alleviate the effects caused by ACR on retinal hyaloid vasculature at 5 dpf. A) Representative confocal images of hyaloid vasculature. White scale bar: 20 µm. B) Quantification of hyaloid vasculature showed significant increasing numbers of branches in *akr1a1a1^+/+^* larvae incubated with 10 µm ACR but rescued by carnosine (dissolved in egg water) and PK11195 (dissolved in DMSO) at 5 dpf, *n* = 11–18. For statistical analysis one‐way ANOVA followed by Tukey's multiple comparisons test was applied. **p < 0.01, ****p < 0.001. NS, not significant. DMSO, dimethylsulfoxide. CAR, carnosine. PK, PK11195.

ACR has been reported as a toxic antioxidant that can cause alterations in various tissues, but whether the alterations in hyaloid vasculature result from reduced *insra/insrb* expression or by ACR directly remains unknown. Therefore, RCS scavenger–l‐carnosine, which forms carnosine‐ACR Michael adducts,^[^ ^35^ ^]^ and hypoglycemic drug PK11195 were selected for the co‐incubation experiment on wild type larvae with ACR. Surprisingly, both l‐carnosine and PK11195 could reverse the alterations in hyaloid vasculature, suggesting ACR leads to alterations in hyaloid vasculature via altered glucose homeostasis instead of direct toxic effects caused by itself (**Figure** **8**). Moreover, the same rescue experiments were also processed in *akr1a1a^−/−^* larvae in which the normalized hyaloid vasculature were observed after the treatment with l‐carnosine and PK11195 (Figure S13, Supporting Information). To sum up, successful reversion of the hyaloid phenotypes by applying hypoglycemic drug and ACR‐scavenger implies that these drugs are potential candidates for treating ACR‐induced vascular alterations.

## 3 Discussion

In this study, we established an Akr1a1a knockout zebrafish model with increased endogenous ACR concentrations for the first time. The further study proved impaired ACR detoxification and increased internal ACR concentration due to Akr1a1a loss induces insulin resistance and impaired glucose homeostasis and subsequently caused abnormal angiogenesis in the hyaloid vasculature in larvae and resulted in angiogenic retina vessels and GBM thickening in adults.

Type 2 diabetes (T2DM) is considered the primary subtype of diabetes, which occupies vast amounts of cases and is characterized by insulin resistance.^[^ ^2^, ^36^ ^]^ With this, finding out the initial trigger leading to insulin resistance becomes more and more essential. More importantly, a better understanding of the drivers in the proceeding procedure of insulin resistance may help diagnose and prevent T2DM early and provide new insight into promising therapeutic approaches. Up to now, several risk factors such as accumulation of ectopic lipid metabolites, activation of the unfolded protein response pathway, and innate immune pathways have been identified as potential pathological pathways of insulin resistance.^[^ ^37^, ^38^ ^]^ Nevertheless, the molecules by which such pathways cause the onset of insulin resistance remained unclear.

To date, MG, a hazardous reactive metabolite, has been identified as the main precursor of advanced glycation end products (AGEs) which is strongly linked to the development of insulin resistance and microvascular complications,^[^ ^9^, ^39^, ^40^, ^41^ ^]^ but a limited number of studies have reported if ACR is involved in the onset of insulin resistance and diabetic complications. Our work in zebrafish firmly supports an elevated endogenous ACR leads to hyperglycemia, identifying a new metabolic intermediate related to diabetes. In zebrafish, we found excess ACR due to the loss of Akr1a1a enzyme system inhibited the expression of insulin receptor a and b, disrupted insulin signaling transduction, and finally resulted in impaired glucose homeostasis and diabetic organ damage. Besides, this study offers a novel animal model with high internal ACR and brings the possibility and convenience for studying physiological functions of ACR in vivo.

ACR is recognized as a side‐product and appears accompanied by diabetic retinopathy and nephropathy due to the disordered lipid peroxidation.^[^ ^11^, ^14^, ^42^ ^]^ This was also observed in our results which exhibited angiogenic retina vessels and thickening GBM in *akr1a1a* mutants consistent with early pathological alterations in diabetic retinopathy and nephropathy.^[^ ^43^, ^44^ ^]^ This raised the hypothesis, whether ACR is the marker or the maker of hyperglycemia and diabetic complications. To address this question, a known hypoglycemic drug PK11195 and ACR scavenger l‐carnosine were utilized to determine if neutralizing ACR or decreasing the glucose level can impede the ACR leading effects. Intriguingly, ACR‐induced hyaloid angiogenic alterations can be reversed by either anti‐hyperglycemic or anti‐ACR treatment. The data now afford several important implications. First, it suggests Akr1a1a as a principle enzyme to detoxify ACR in zebrafish and AKR family is involved in glucose metabolism and diabetes. Second, it implies the accumulated ACR as the upstream metabolite triggering organic alterations via adjusting glucose homeostasis. Last, in addition to being an escort of diabetic complications, this study identified ACR, at least in zebrafish, as an effective inducer causing insulin resistance and diabetic complications, providing a new therapeutic target for diabetic complications treatment.

In clinical studies, elevated free‐state ACR or ACR‐adducts have been identified as distinct features in patients with diabetes and diabetic complications. It was found that ACR‐lysine adduct (FDP‐lysine) accumulated in both type 1 and type 2 diabetic patients’ urine and even more in diabetic patients with microalbuminuria.^[^ ^18^, ^19^ ^]^ Additionally, in end‐stage renal disease, the FDP‐lysine level of those patients with type 2 diabetes was significantly higher than the non‐DM group,^[^ ^21^ ^]^ which implies FDP‐lysine has a strong connection with diabetic nephropathy. Furthermore, it was suggested that FDP‐lysine level could be utilized as a biomarker for the severity of diabetic retinopathy.^[^ ^22^ ^]^ However, the crucial role of ACR in the onset of T2DM required further investigations. Based on our study, it would be a promising strategy to dynamically monitor endogenous ACR concentration in clinics for filtering the people with the pre‐diabetic state, early diagnosing patients with insulin resistance, and improving long‐term prognosis. Lastly, this study also affords an alternative strategy for diabetes treatment. General therapy applying hypoglycaemic agents and insulin sensitizer require patients to take medications for their whole life to achieve ideal and stable glucose control.^[^ ^45^, ^46^ ^]^ Whether it would be practical to treat diabetes and diabetic complications by using ACR scavenger, such as carnosine, deserves further study.

Although this study successfully illustrated ACR elevation contributes to impaired glucose homeostasis, there are some limitations. First, although it has been confirmed that ACR cause reduced *insra/insrb* expression, the detailed mechanism remained unknown. Second, whether the impaired glycolysis appearing in *akr1a1a^−/−^* adults caused by reduced PFK activity results from insulin resistance or partially caused by accumulated internal ACR is still controversial. Finally, clinical studies are more than essential to determine the sensitivity and specificity of ACR in predicting insulin resistance and pre‐diabetic state.

In conclusion, this study provided patent evidence for the contribution of poor ACR detoxification and subsequently increased ACR concentration to the development of impaired glucose homeostasis via insulin receptor signaling dysfunction in *akr1a1a* mutants, providing a novel direction for future research regarding diabetic pathophysiology and therapy.

## 4 Experimental Section

### Zebrafish Husbandry and Zebrafish Lines

Zebrafish lines, *Tg(fli1:EGFP)* was raised and staged as described under standard husbandry environment.^[^ ^33^, ^47^ ^]^ Embryos/larvae were kept in E3 media at 28.5 °C with/without PTU (2.5 mL in 25 mL) to suppress pigmentation formation. Adult zebrafish were kept under 13 h light/11 h dark cycle and fed with living shrimps in the morning and fish flake food in the afternoon. All experimental interventions on animals were approved by the local government authority, Regierungspräsidium Karlsruhe and by Medical Faculty Mannheim (license no: G‐98/15 and I‐19/02) and carried out in accordance with the approved guidelines. Age of adult male zebrafish was from 9 to 16 months. Both sexes were only included for the blood glucose measurements and RT‐qPCR.

### Mutant Generation

The CRISPR target site for *akr1a1a* was identified and selected using ZiFiT Targeter 4.1. The *akr1a1a*‐CRISPR oligonucleotides were synthesized by Sigma‐Aldrich. The oligonucleotides were cloned into the pT7‐gRNA plasmid (Addgene). BamHI‐HF (Biolabs) was used for linearization. Cas9 mRNA was synthesized from pT3TS plasmid (Addgene) after linearizing with XbaI (Biolabs). Plasmids were purified with a PCR purification kit (Qiagen). CRISPR gRNA in vitro transcription was done by the T7 MEGAshortscript kit and mMESSAGE MACHINE T3 kit for Cas9 mRNA (Invitrogen). Purification of RNA after TURBO DNAse treatment was done with the MiRNeasy Mini (gRNA) and RNeasy Mini (Cas9 mRNA) kits (Qiagen). *Akr1a1a* gRNA and Cas9 mRNA (150pg nL^−1^) was mixed with KCl (0.1 m). The RNA mixture (1 nL) was injected directly into one‐cell embryos. For genotyping, PCR‐products of genomic DNA were used for Sanger sequencing (Table S1, Supporting Information).

### Morpholinos

Morpholinos including SB‐*insra*‐MO, SB‐*insrb*‐MO, and Control‐MO (Table S2, Supporting Information) were designed and produced by GENE TOOLS, LLC. All morpholinos were diluted to 2 µg µL^−1^ with 0.1 m KCl. One nanoliter of morpholino was injected into the yolk sack of one‐cell stage of the embryos as previously described.^[^ ^48^ ^]^ Morpholinos and the genotyping primers for zebrafish *insra/insrb* morpholinos are listed in Table S3, Supporting Information.

### Antibody Generation

For Akr1a1a antibody generation, peptide (RLIESFNRNERFII‐C) was designed, synthesized, and coupled to KLH (Keyhole Limpet Hemocyanin) by PSL GmbH, Heidelberg, Germany and subsequently injected into guinea pigs for immunization following standard procedures from GPCF Unit Antibodies, DKFZ Heidelberg, Germany.

### Western Blot Analysis

For western blot analysis, larvae/adult organs were taken and incubated for 10 min with 2 mm Natrium‐Vanadate in 1× PBS on ice to inhibit phosphatases and then lysed in NP40 lysis buffer (150 mmol L^−1^ NaCl, 50 mmol L^−1^ Tris‐HCl, pH 7.4, 1% NP40, 10 mmol L^−1^ EDTA, 10% glycerol, and protease inhibitors), followed by homogenization with TissueLyser II (Qiagen) and incubation on ice for 30 min on a shaker. The supernatant containing the protein lysate was diluted 5:1 with Laemmli sample buffer and boiled at 95 °C for 5 min, separated via SDS‐PAGE, and then transferred to a nitrocellulose membrane for antibody incubation (anti‐Akr 1a1a antibody 1:1000, anti‐Actin antibody A2228 from Sigma‐Aldrich, 1:1000, AKT antibody 9272S from CST, 1:1000, P‐AKT antibody 4060P from CST, 1:1000), secondary HRP‐conjugated antibodies 1:1000 (for *β*‐actin: rabbit anti‐goat, P0160, Dako; for Akr1a1a: goat anti‐guinea pig, ABIN101281, antibodies‐online.com, for P‐AKT and AKT, Goat anti‐Rabbit, P0448, Dako). Visualization by enhanced chemiluminescence (ECL) was acquired after incubation with HRP (Horseradish Peroxidase) substrate.

### Preparation of Adult Zebrafish and Blood Glucose Measurement

Adult zebrafish were transferred into single boxes one day before and fasted overnight. Sixteen hours later, fish were tested directly or fed with 0.5 g flake food for 1 h followed by refreshing water and 1 h postprandial experiment. Afterward, fishes were euthanized with 0.025% tricaine until the operculum movement disappeared entirely. Then blood was extracted from caudal vessels and blood glucose was measured by a glucometer. Later on, fishes were sacrificed and transferred into experimental platform covered with ice‐cold PBS. Organs were isolated, weighed, snap frozen in liquid nitrogen and stored at −80 °C for metabolomics, RT‐qPCR and ELISA analysis. Age of adult zebrafish was from 10 to 12 months. The whole fish head was transferred into 4% PFA/PBS for 24 h at 4 °C for further retinal vasculature analysis.

### Microscopy and Analysis of Vascular Alterations in Larvae and Adults

For imaging of the zebrafish retinal hyaloid vasculature, *Tg(fli1: EGFP)* larvae were anesthetized in 0.0003% tricaine at 120 hpf, and fixed in 4% PFA/PBS overnight at 4 °C. Fixed larvae were washed three times for 10 min per time in double distilled water (ddH_2_O) and incubated for 90 min at 37 °C in 0.5% Trypsin/EDTA solution (25200‐056, Gibco) buffered with 0.1 m TRIS (Nr. 4855.3, Roth) dilution and adjusted to pH 7.8 with 1 m HCl solution. Larval hyaloid vasculature was dissected under a stereoscope and displayed in PBS for visualization according to Jung's protocol.^[^ ^49^ ^]^ Confocal images for phenotype evaluation were acquired using a confocal microscope (DM6000 B) with a scanner (Leica TCS SP5 DS) utilizing a 20 × 0.7 objective, 1024 × 1024 pixels, 0.5 µm Z‐steps. Vascular cross points of blood vessels were regarded as “branches” and small new blood vessels were counted and addressed as “sprouts” within the circumference of the hyaloid per sample.

For imaging of the zebrafish adult retinal vasculature, retina dissection and analysis were performed as recently described.^[^ ^50^ ^]^ In brief, PFA fixed heads from adult zebrafish were transferred to agarose platform covered with 1× PBS and eyes were removed from the head as the first step. Retina was detached from eye and washed twice with 1× PBS. Washed retina was immersed in mounting media and covered with a cover slide. Images were taken by using DM6000 B confocal microscope with Leica TCS SP5 DS scanner. Parameter: 600 Hz, 1024 × 1024 pixels and 1.5 µm thick of z‐stacks were utilized. Quantification of branch points and sprouts was performed by using GIMP and ImageJ in squares of 350 × 350 µm^2^.

### Analysis of Kidney Morphology

Kidneys for EM study were fixed for at least 2 h at room temperature in 3% glutaraldehyde solution in 0.1 m cacodylate buffer pH 7.4, cut into pieces of ≈1 mm^3^, washed in buffer, post fixed for 1 h at 4 °C in 1% aqueous osimium tetroxide, rinsed in water, dehydrated through graded ethanol solutions, transferred into propylene oxide, and embedded in epoxy resin (glycidether 100). Semithin and ultrathin sections were cut with an ultramicrotome (Reichert Ultracut E). Semithin sections of 1 µm were stained with methylene blue. 60–80 nm ultrathin sections were treated with uranyl acetate and lead citrate, and examined with an electron microscope JEM 1400 equipped with a 2K TVIPS CCD Camera TemCam F216. Kidneys were fixed in 10% buffered formalin for Periodic acid‐Schiff staining, removed, routinely embedded in paraffin, and cut into 4 µm‐thick sections. For quantification of GBM on EM sections, up to 15 images were analyzed per genotype.

### Pharmacological Treatment of Zebrafish Embryos/Larvae

Fertilized zebrafish embryos were transferred into 6‐well plate, around 30 embryos per well with 5 mL eggwater. At 24 hpf the chorion of zebrafish embryos was removed using sharp tweezers and 0003% PTU was added to the eggwater. For ACR intervention and rescue experiments, 10 µm ACR (S‐11030F1; CHEM SERVICE), 10 µm PK11195 (C0424; Sigma‐Aldrich), 10 mm l‐Carnosine (C9625; Sigma‐Aldrich) treatments were started from 24 hpf and continued until the end. Medium was refreshed daily.

### Whole‐Body Glucose Determination in Zebrafish Larvae

Zebrafish larvae were collected at 5 dpf and snap frozen. Approximately 20–25 larvae per clutch were homogenized in glucose assay buffer by the ultrasonic homogenizer, 90% intensity, and 15 s for 2 times. Glucose content was determined according to manufacturer's instruction (Glucose Assay Kit, CBA086, Sigma‐Aldrich).

### ACR Determination in Zebrafish Larvae and Liver

Zebrafish larvae at 96 hpf and adults’ liver were collected and snap frozen. Approximately 40–50 larvae per clutch were homogenized in 1× PBS with by the ultrasonic homogenizer, 90% intensity, 15 s for 2 times. Protein‐bound ACR was determined according to manufacturer's instruction (Acrolein ELISA Kit, MBS7213206, MyBioSource Inc).

### Enzyme Activity Assay

At 96 hpf, around 50 zebrafish larvae per measurement were anaesthetized with 0.003% tricaine and snap frozen. ALDH activity was assayed at 25 °C in 75 mm Tris‐HCl (pH 9.5) containing 10 mm DL‐2‐ amino‐1propanol, 0.5 mm NADP, and 2 mm MG by measuring the rate of NADP formation at 340 nm. Glo1‐activity was determined spectrophotometrically monitoring the change in absorbance at 235 nm caused by the formation of S‐D‐lactoylglutathione. AKR activity was determined by measuring the rate of reduction of NADPH at 340 nm, pH 7.0, and 25 °C. The assay mixture contained 100 mm potassium phosphate, 10 mm DL‐Glyceraldehyde/5 mm ACR, and 0.1 mm NADPH. The glucokinase activity was determined based upon the reduction of NAD through a coupled reaction with glucose‐6‐phosphate dehydrogenase and was measured spectrophotometrically by the increase in absorbance at 340 nm.^[^ ^51^ ^]^ The phosphofructoskinase activity assay was based upon the oxidation of NADH through a coupled reaction with aldolase, triosephosphate isomerase, and glyceraldehyde‐3‐phosphate dehydrogenase. Activity was determined by measuring the decrease in absorbance at 340 nm.^[^ ^52^ ^]^ The pyruvatekinase activity assay was based upon the oxidation of NADH through a coupled reaction with l‐lactic dehydrogenase and was determined spectrophotometrically by increase the decrease in absorbance at 340 nm.^[^ ^53^ ^]^

### Detection of S‐Nitrosylation

S‐Nitrosylation was detected by using the Biotin Switch Assay Kit (Abcam, ab236207) as described previously.^[^ ^26^ ^]^

### MG and Glyoxal Measurement

At 96 hpf, around 50 zebrafish larvae per measurement were anaesthetized with 0.003% tricaine and snap frozen. MG and glyoxal were measured as previously described.^[^ ^48^ ^]^

### Metabolomic Analysis

Detection was done in cooperation with the Metabolomics Core Technology Platform from the Centre of Organismal Studies Heidelberg. 50 zebrafish larvae at 96 hpf per measurement or livers were snap frozen in liquid nitrogen. Adenosine compounds, thiols, free amino acids, fatty acids, and primary metabolites were measured as previously described.^[^ ^9^ ^]^

### Reverse‐Transcription Quantitative Polymerase Chain Reaction Analysis (RT‐qPCR)

Total RNA was isolated from *TG(fli1:EGFP)* zebrafish larvae/adult organs at different time points using the RNeasy Mini Kit following the manufacturer's protocol (Qiagen). cDNA Synthesis and qPCR reaction were proceeded as previously described.^[^ ^26^ ^]^ Primers are listed in Table S2, Supporting Information.

### RNA‐seq Analysis

Total RNA was isolated from *akr1a1a^+/+^*, *akr1a1a^−/−^* and *akr1a1a^+/+^* with ACR treatment larvae at 120 hpf. Library construction and sequencing were performed with BGISEQ‐500 (Beijing Genomic Institution, www.bgi.com, BGI). Gene expression analysis were conducted by the Core‐Lab for microarray analysis, center for medical research (ZMF). Quality control and data analysis were performed as described previously.^[^ ^48^ ^]^ The RNA‐Seq datasets produced in this study are available at GEO (Gene Expression Omnibus, NIH) under the accession number: https://www.ncbi.nlm.nih.gov/geo/query/acc.cgi?acc = GSE168786.

### Protein Sequence Alignment

The amino acid sequences of the Akr1a1a proteins from zebrafish (Q6AZW2_DANRE), human (AK1A1_HUMAN), and mouse (AK1A1_MOUSE) were accessed from the UniProt Database (http://www.unip rot.org/). For the comparison, the genes were selected and aligned with the UniProt‐own alignment tool (http://www.uniprot.org/align/).

### Software

For zebrafish *akr1a1a* exon/intron region, amino acid and other schematic diagrams generation, websites of Ensembl (https://www.ens embl.org/), Biorender (https://biorender.com/) were used. Analysis of retinal vasculature was carried out by using LAS AF Lite Software from Leica for taking screenshots, Gimp for image cutting and ImageJ for quantification. The “GCMS solution” software (Shimadzu) was used for data processing of the GC/MS analysis.

### Statistical Analysis

Sample size for all experiments was more than three independent biological replicates. Data were displayed as mean with standard deviation. Statistical significance between different groups was analyzed using two‐paired Student's *t*‐test or one‐way ANOVA (followed by Tukey‘s multiple comparisons) in GraphPad Prism 6.01 or 8.3.0. *p*‐values of 0.05 were considered as significant: *p < 0.05, **p < 0.01, ***p < 0.001, ****p < 0.0001.

### Material Requests

The anti‐Akr1a1a antibody and the akr1a1a zebrafish mutant generated in this study are available from the corresponding author with a completed Materials Transfer Agreement.

## Conflict of Interest

The authors declare no conflict of interest.

## Author Contributions

H.Q. performed experiments, analyzed data, and wrote the manuscript. F.S. and K.B. generated the *akr1a1a* mutant and performed the analysis of vascular alterations. X.L. performed *akr1a1b* related experiments. C.S. performed RNA‐Seq analysis. J.M., T.F., and X.Q. performed biochemical experiments and analyzed data. C.T.T., N.V., and I.H. performed histological analyses including electron microscopy of adult kidneys. E.H. performed metabolome experiments and analyzed data. R.H. and P.P.N. gave conceptual and technological advice. J.K. conceived and designed the study and wrote the manuscript. J.K. is the guarantor of this work and, as such, has full access to all the data in the study and takes responsibility for the integrity of the data and the accuracy of the data analysis.

## Supporting information

Supporting Information

## Data Availability

The data that support the findings of this study are available from the corresponding author upon reasonable request. The RNA‐seq data that support the findings of this study are openly available at GEO (Gene Expression Omnibus, NIH) under the accession number: https://www.ncbi.nlm.nih.gov/geo/query/acc.cgi?acc=GSE168786.
