# Supplementary material for: Reduced Acrolein Detoxification in akr1a1a Zebrafish Mutants Causes Impaired Insulin Receptor Signaling and Microvascular Alterations
Source: Adv Sci (Weinh). 2021 Jul 18;8(18):2101281. doi: 10.1002/advs.202101281 (PMC8456208; doi:10.1002/advs.202101281)
Supplement: Supplementary file 1 — Supporting Information [file ADVS-8-2101281-s001.pdf]

© 2021 Wiley-VCH GmbH

ADVANCED SCIENCE
Open Access

Supporting Information

for *Adv. Sci.*, DOI: 10.1002/advs.202101281

Reduced Acrolein Detoxification in *akr1a1a* Zebrafish Mutants Causes Impaired Insulin Receptor Signaling and Microvascular Alterations

*Haozhe Qi, Felix Schmöhl, Xiaogang Li, Xin Qian, Christoph T. Tabler, Katrin Bennewitz, Carsten Sticht, Jakob Morgenstern, Thomas Fleming, Nadine Volk, Ingrid Hausser, Elena Heidenreich, Rüdiger Hell, Peter Paul Nawroth and Jens Kroll**

Supporting Information

Reduced Acrolein Detoxification in *akr1a1a* Zebrafish Mutants Causes Impaired Insulin Receptor Signaling and Microvascular Alterations

Haozhe Qi[1,2], Felix Schmöhl[1], Xiaogang Li[1], Xin Qian[1], Christoph T. Tabler[1], Katrin Bennewitz[1], Carsten Sticht[3], Jakob Morgenstern[4,5], Thomas Fleming[4,5], Nadine Volk[6], Ingrid Hausser[7], Elena Heidenreich[8], Rüdiger Hell[8], Peter Paul Nawroth[4,5,9] and Jens Kroll[1]*

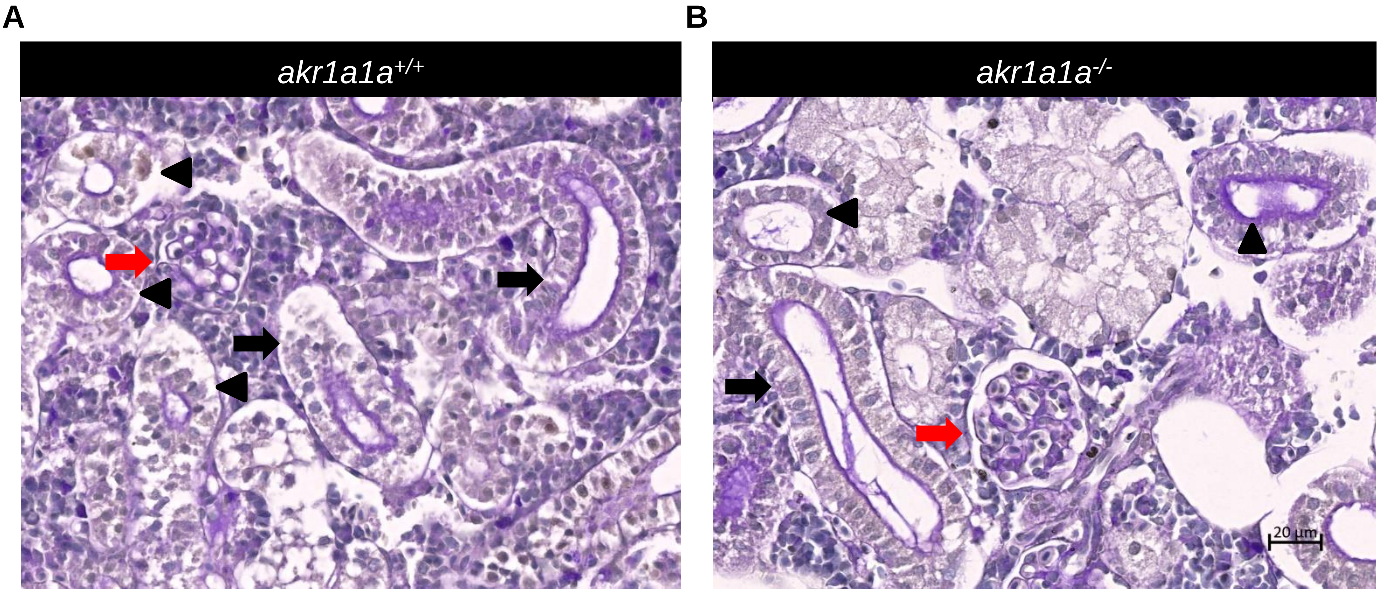

Figure.S1 Unaltered renal morphology in adult *akr1a1a*$^{-/-}$ mutants.

(A,B). Representative Periodic acid–Schiff (PAS) staining showed normal gross structure of *akr1a1a*$^{+/+}$ *and akr1a1a*$^{-/-}$ kidneys. Red arrow: glomeruli. Black arrow: proximal tubule. Black triangle: distal tubule. Black scale bar: 20 µm.

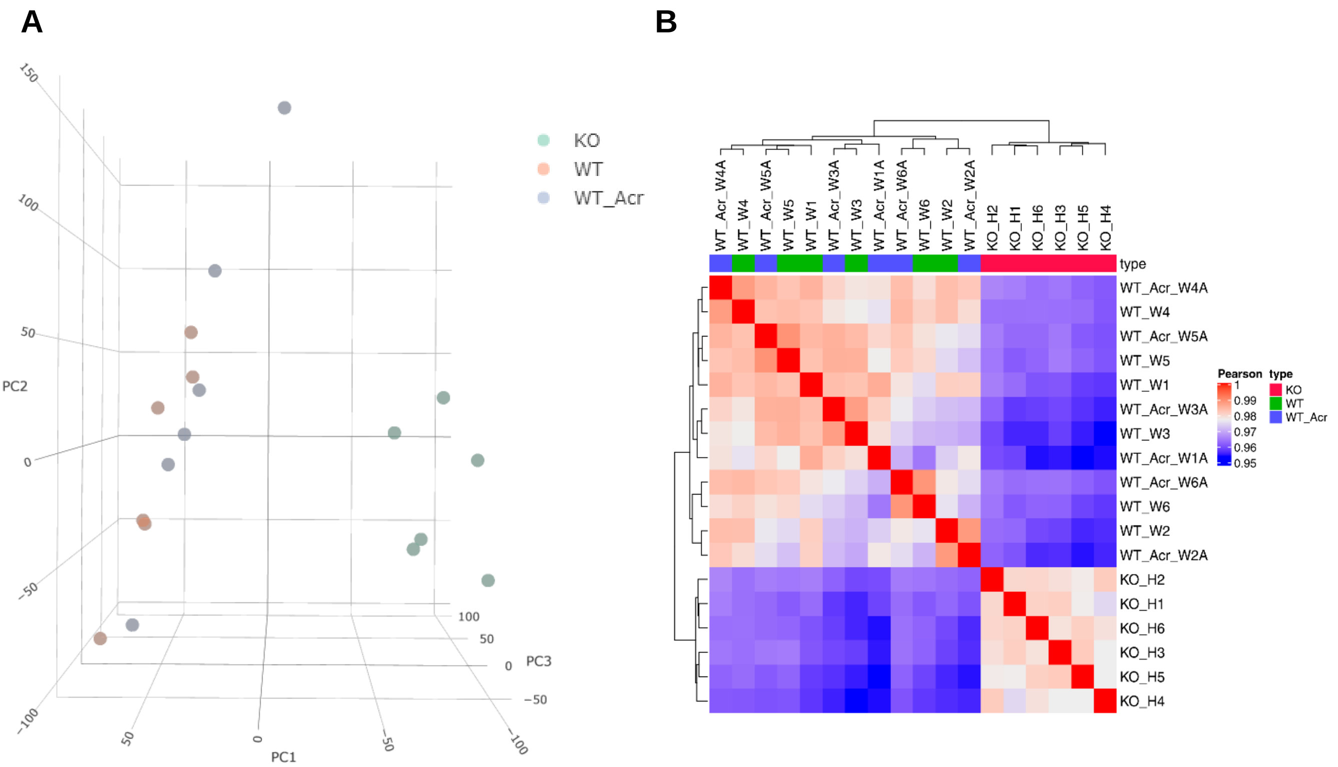

Figure.S2 An overview of RNA Sequencing Results.

(A). Results of the quality control in gene expression analysis between *akr1a1a*$^{+/+}$, *akr1a1a*$^{-/-}$ and *akr1a1a*$^{+/+}$ with ACR treatment zebrafish larvae at 120 hpf. Principal component 1, 2 and 3 are on the axis. The plots showed the *akr1a1a*$^{-/-}$ (n = 6) in green, *akr1a1a*$^{+/+}$ (n = 5) in red and *akr1a1a*$^{+/+}$ with ACR treatment in blue. (B). Heatmaps of each samples showed comparable property between *akr1a1a*$^{-/-}$, *akr1a1a*$^{+/+}$ and *akr1a1a*$^{+/+}$ with ACR treatment zebrafish larvae.

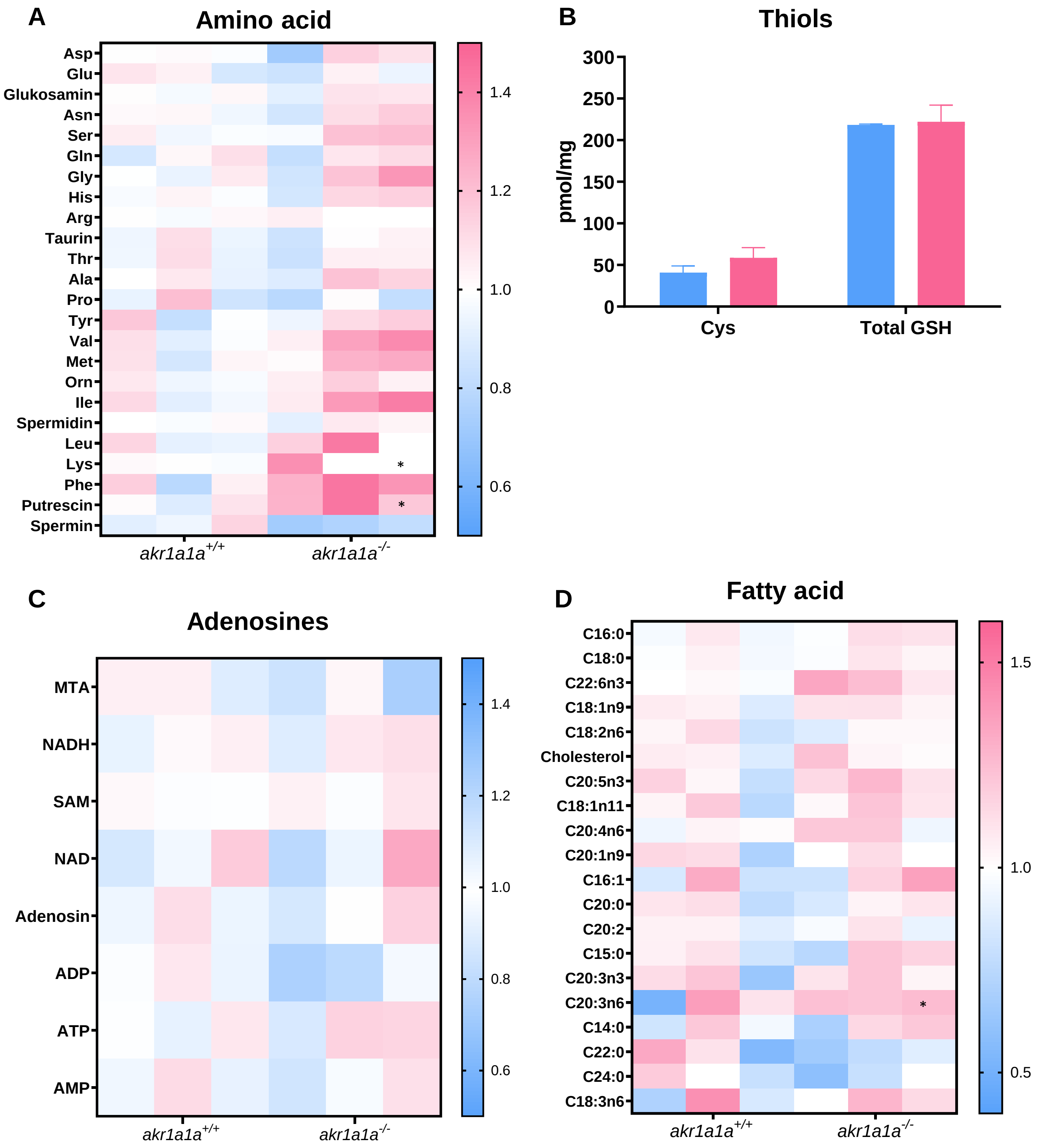

Figure.S3 Metabolomic profiling displayed minor alterations between *akr1a1a*$^{+/+}$ and *akr1a1a*$^{-/-}$ zebrafish larvae at 96 hpf. (A). Lysine and putrescin were significantly increased in *akr1a1a*$^{-/-}$ mutants. Leucine and phenylalanine displayed increasing tendency while spermin displayed a decreasing tendency in *akr1a1a*$^{-/-}$ mutants. n = 3 clutches with 50 larvae. (B). Cysteine and GSH were unaltered in *akr1a1a*$^{-/-}$ mutants, n = 3 clutches with 50 larvae. (C). Adenosines were unaltered in *akr1a1a*$^{-/-}$ mutants , n = 3 clutches with 50 larvae. (D). C20:3n6 was increased in *akr1a1a*$^{-/-}$ mutants, n = 3 clutches with 50 larvae. For statistical analysis Student's t-test was applied; *p<0.05.

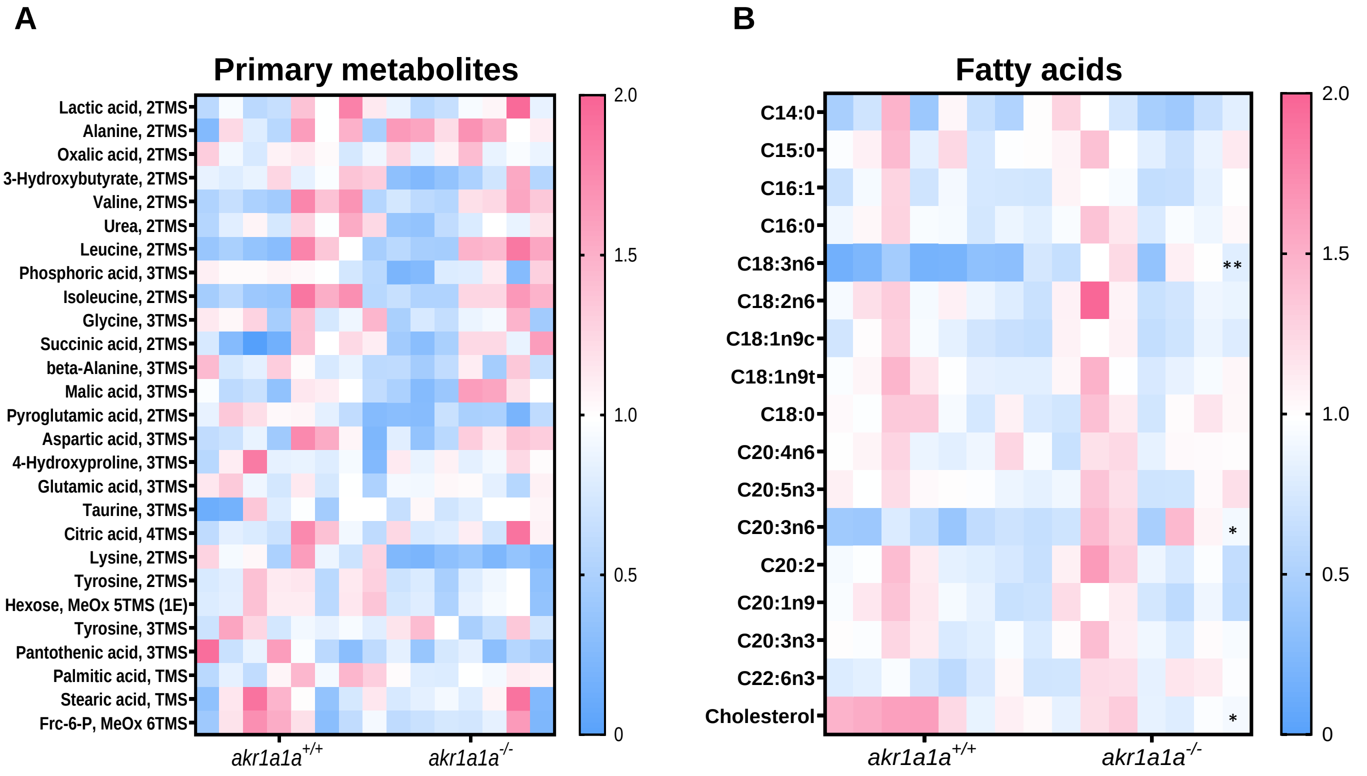

Figure.S4 Metabolomic profiling displayed minor alterations between adult *akr1a1a*$^{+/+}$ and *akr1a1a*$^{-/-}$ zebrafish livers.
(A). Primary metabolites displayed non-significant alterations in *akr1a1a*$^{-/-}$ mutants, n = 7-8. (B). C20:3n6 and C18:3n6 were increased while cholesterol was decreased significantly in *akr1a1a*$^{-/-}$ mutants, n = 7-8. For statistical analysis Student's t-test was applied; *p<0.05. ** p<0.01.

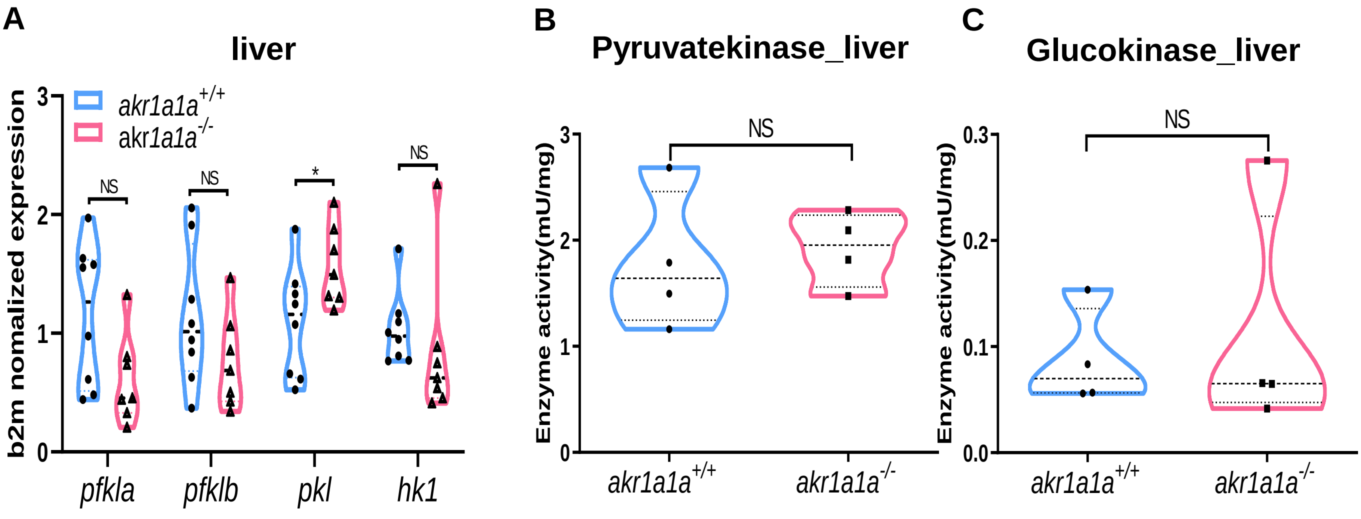

Figure.S5 Glycolytic enzyme expression and activity in adult *akr1a1a*$^{-/-}$ livers.
(A). *Pfk* and *hk1* mRNA expression showed decreasing tendency while *pk* expression increased in *akr1a1a*$^{-/-}$ livers. n = 6. (B-C). PK and GK enzyme activity were unaltered in *akr1a1a*$^{-/-}$ livers. n = 4. For statistical analysis Student's t-test was applied. *p<0.05. NS, not significant. Pfk, Phosphofructokinase. Pk, Pyruvate kinase. Hk, Hexokinase. GK, Glucokinase.

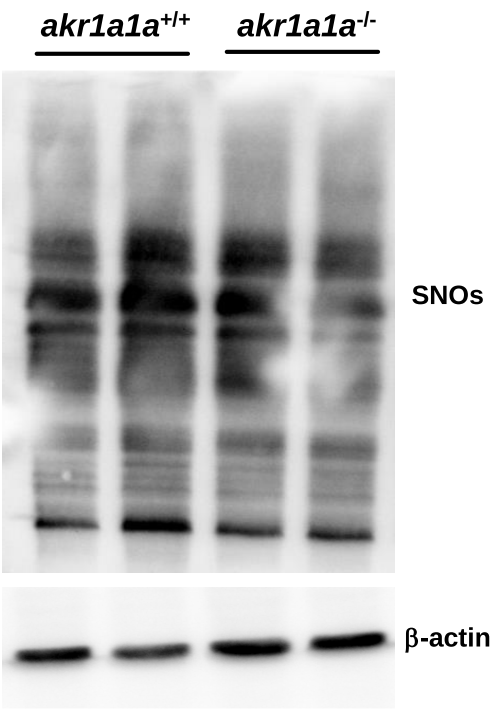

Figure.S6 S-nitrosylated proteins were unaltered in *akr1a1a*$^{-/-}$ livers
Western blot showed unchanged S-nitrosylated proteins (SNOs) in *akr1a1a*$^{-/-}$ livers. β-actin served as loading control.

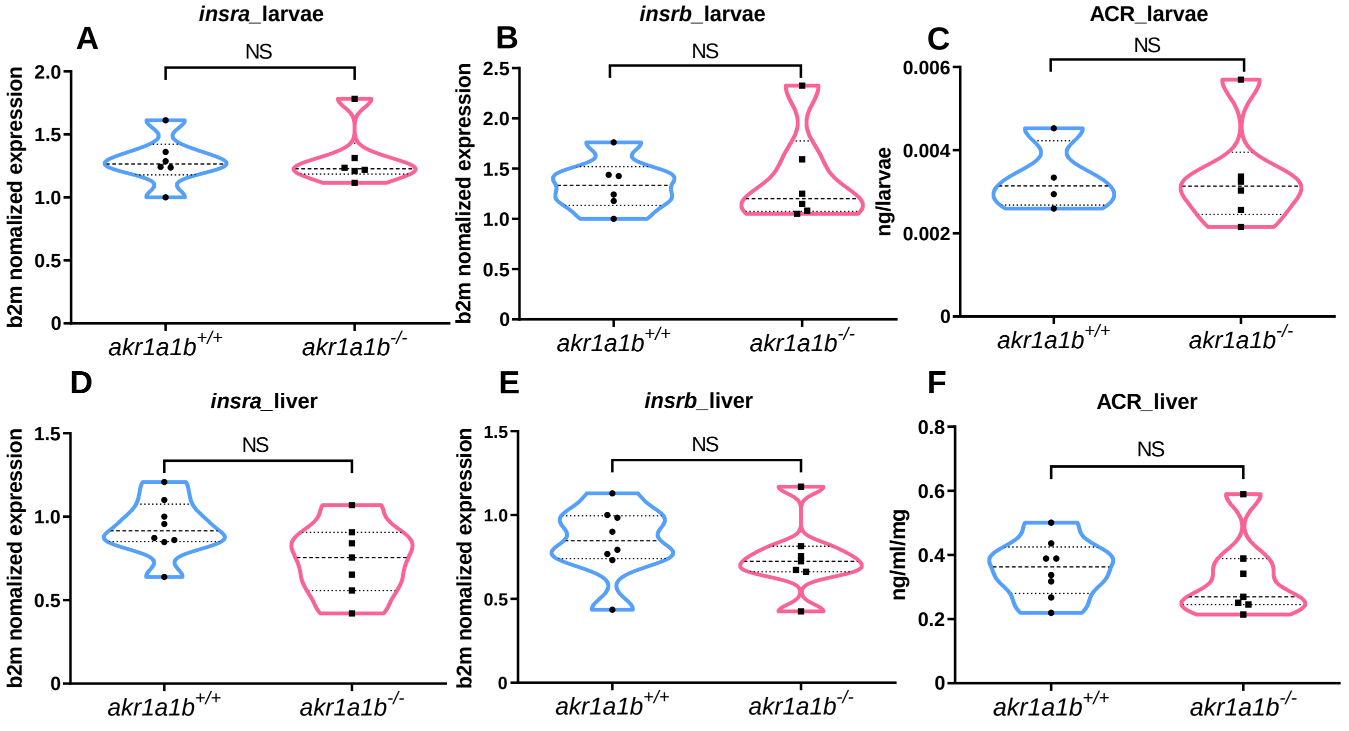

Figure.S7 *insra/insrb* expression and ACR concentrations were unaltered in *akr1a1b* mutants.
(A,B). *insra* and *insrb* mRNA expression were unaltered in *akr1a1b*$^{-/-}$ larvae at 5dpf. n = 6 clutches with 30 larvae. (C). ACR was unchanged in *akr1a1b*$^{-/-}$ larvae. n = 4-6 clutches with 50 larvae. (D,E). *insra* and *insrb* mRNA expression were unaltered in *akr1a1b*$^{-/-}$ liver. n = 7-8. (F). ACR was unchanged in *akr1a1b*$^{-/-}$ liver. n = 7-8. For statistical analysis Student's t-test was applied. NS, not significant.

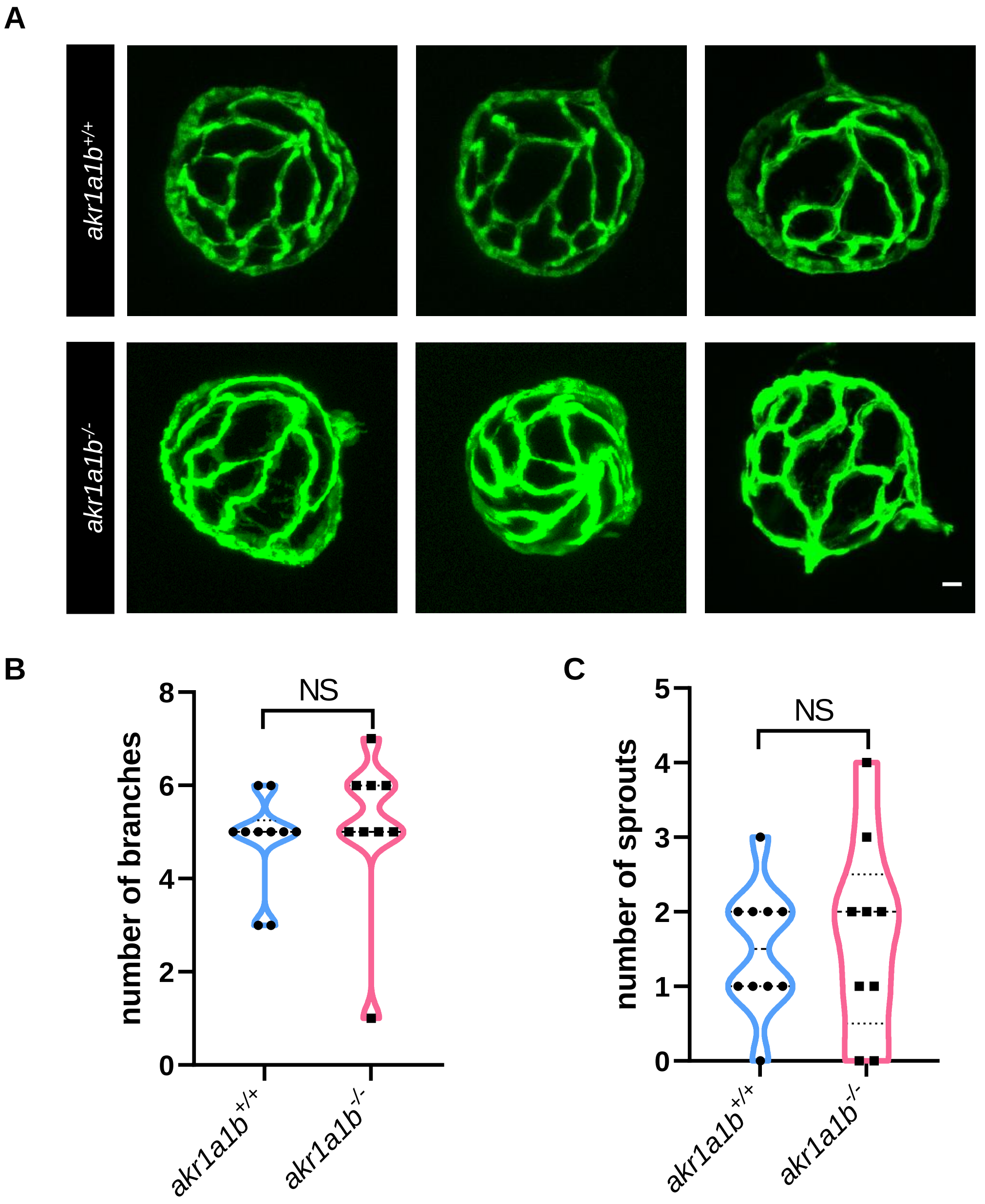

Figure.S8 Hyaloid vasculature is unaltered in *akr1a1b*$^{-/-}$ larvae at 5dpf.
(A). Representative confocal images of hyaloid vasculature. White scale bar: 20 μm. (B,C). Quantification of hyaloid vasculature showed unalterted branches and sprouts in *akr1a1b*$^{-/-}$ larvae at 5dpf. n = 9-10. For statistical analysis Student’s t-test was applied. NS, not significant.

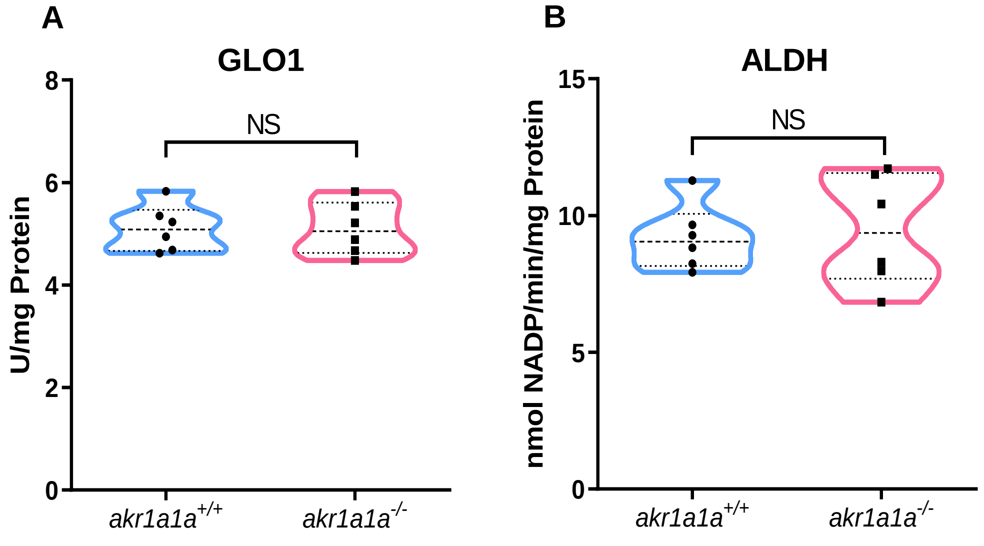

Figure.S9 Glo1 and ALDH enzyme activity were unaltered in *akr1a1a*$^{-/-}$ larvae.
(A,B). Glo1 and ALDH enzyme activities were unaltered in *akr1a1a*$^{-/-}$ larvae. n = 6 clutches with 50 larvae. n = 6 clutches with 50 larvae. For statistical analysis Student's t-test was applied. NS, not significant.

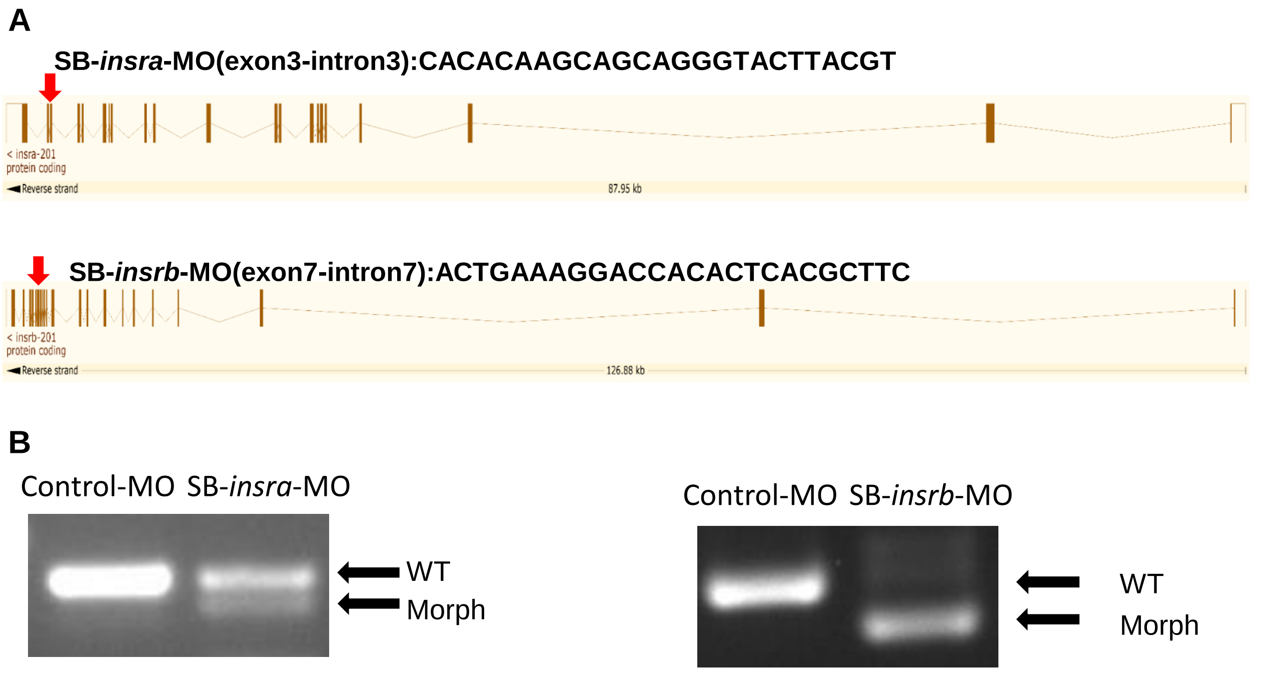

Figure.S10 *Insra/insrb* morpholino design and validation.
(A). SB-*insra*-MO and SB-*insrb*-MO targeting exon3-intron3 and exon7-intron7 junctions of *insra/insrb,* respectively. (B). Validation of splice-blocking morpholinos: SB-*insra*-MO and SB-*insrb*-MO. RT-PCR of Control-MO, SB-*insra*-MO and SB-*insrb*-MO injected larvae showed wild type and generation of morphant *insra/insrb* signals at 24 hpf. 2 ng of morpholinos: Control-MO, SB-*insra*-MO and SB-*insrb*-MO were injected into the one-cell stage of zebrafish embryos, respectively. WT, wild type; MO, morpholino; Morph: morphant.

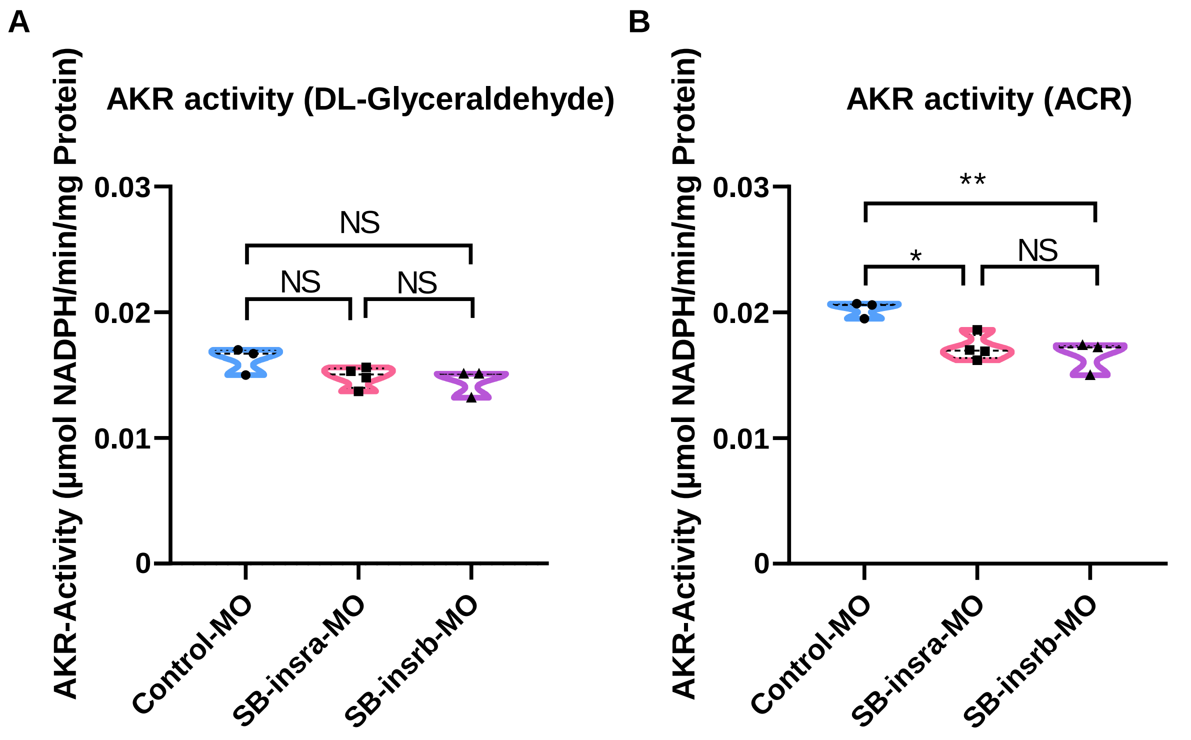

Figure.S11 Reduced AKR activity using ACR as substrate in *insra/insrb* morphants.
(A). *insra/insrb* morphants showed unaltered AKR enzyme activity (DL-Glyceraldehyde served as substrate) measured by spectrophotometric analysis in zebrafish lysates at 96 hpf; n = 3-4 clutches with 50 larvae. (B). *insra/insrb* morphants showed decreased AKR enzyme activity (ACR served as substrate) measured by spectrophotometric analysis in zebrafish lysates at 96 hpf; n = 3-4 clutches with 50 larvae. For statistical analysis one-way ANOVA followed by Tukey's multiple comparison test was applied, *p<0.05. **p<0.01. NS, not significant.

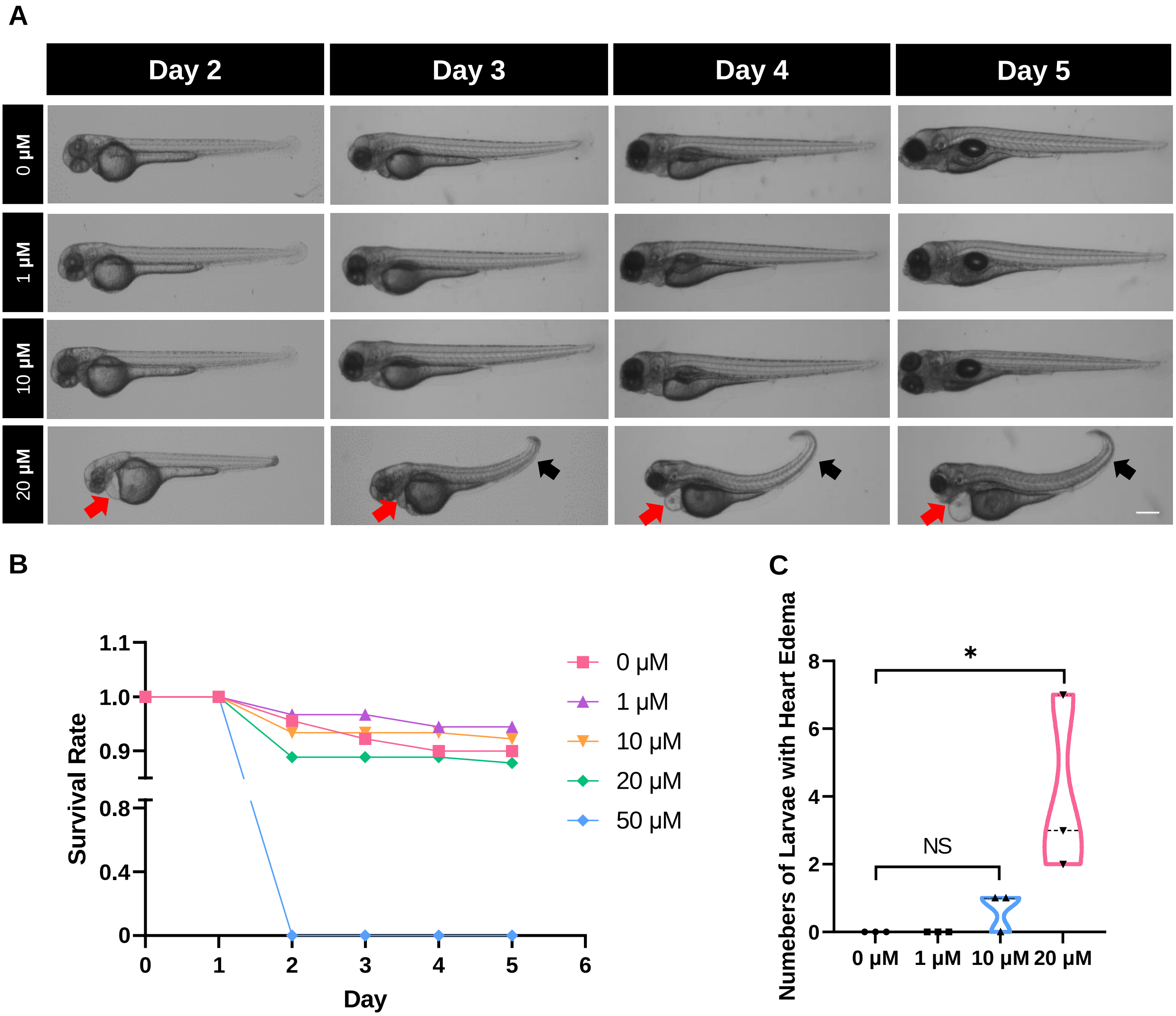

Figure.S12 High concentrations of ACR harm development of zebrafish larvae.
(A). Representative microscopic images showed destructive effects of 20µM on wildtype zebrafish larvae. Red arrow: heart edema. Black arrow: curly tail. Scale bar: 200 µm. (B). Survival rate showed strong lethality of 50µM ACR. (C). The number of larvae with heart edema increased significantly with 20µM ACR treatment. For statistical analysis one-way ANOVA followed by Tukey's multiple comparisons test was applied. *$p < 0.05$. NS, not significant.

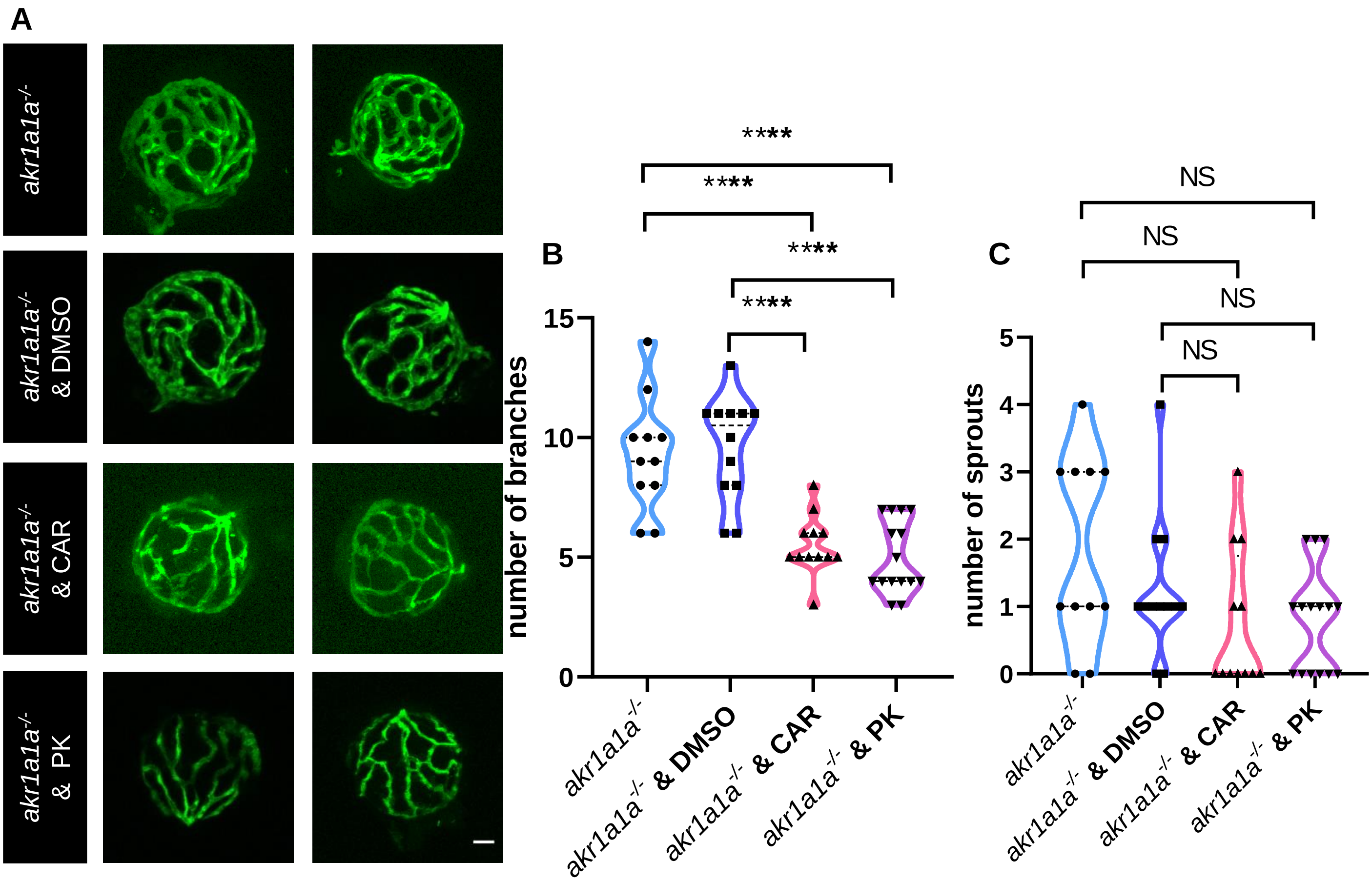

Figure.S13 Carnosine and PK11195 can alleviate the angiogenic retinal hyaloid vasculature in *akr1a1a*$^{-/-}$ larvae at 5dpf.
(A). Representative confocal images of hyaloid vasculature. White scale bar: 20 µm. (B,C). Quantification of hyaloid vasculature showed Carnosine (dissolved in egg water) and PK11195 (dissolved in DMSO) can reverse the increasing numbers of branches in *akr1a1b*$^{-/-}$ larvae at 5dpf. n = 11-15. For statistical analysis one-way ANOVA followed by Tukey's multiple comparisons test was applied. ****p<0.0001. NS, not significant. DMSO, dimethylsulfoxid. CAR, carnosine. PK, PK11195.

**CRISPR-construct**	**Oligonucleotide sequence(5‘ to 3‘)**
akr1a1a-CRISPR-for	TAGGTCAGAGGATGCCAACGGT
akr1a1a-CRISPR-rev	AAACACCGTTGGCATCCTCTGA
**Genotyping primer**	**Primer sequence (5‘ to 3‘)**
akr1a1a-Crisp-Genotype-for	TCATTTGGGCAGGAAAACGT
akr1a1a-Crisp-Genotype-rev	GTAGCCACAGTCTAAAGCTGC

Table S1. CRISPR construct and genotyping primers for zebrafish *akr1a1a*.

qPCR primer name	Primer sequence
b2m-qPCR-for	ACTGCTGAAGAACGGACAGG
b2m-qPCR-rev	GCAACGCTCTTTGTGAGGTG
insa-qPCR-for	GGTCGTGTCCAGTGTAAGCA
insa-qPCR-rev	GGAAGGAAACCCAGAAGGGG
Insra-qPCR-for	AGAGGCCAGCGAGCTCTAC
Insra-qPCR-rev	CACTTGTGTGGGGGCTCT
Insrb-qPCR-for	GCCTCTGCGGATCACTACAT
Insrb-qPCR-rev	CTCCTGCGTGGTCTTGAAC
pfkla-qPCR-for	ACTGCCACTCCAGCGTTAAA
pfkla-qPCR-rev	CAGAGCTGGAGTTCACCCTC
pfklb-qPCR-for	GCCGTTCAACATTCACGACC
pfklb-qPCR-rev	TGCAGTCGAACACTCCTTGG
pkl-qPCR-for	TCCTGGAGCATCTGTGTCTG
pkl -qPCR-rev	GTCTGGCGATGTTCATTCCT
hK1-qPCR-for	ATGATAGCGGCACAGCTTCT
hk1-qPCR-rev	GTTGGTGTCTCGTGCCAATC

Table S2. qPCR primers.

**Morpholinos**	**sequence**
SB-*insra*-MO	CACACAAGCAGCAGGGTACTTACGT
(exon3-intron3 junction)	
SB-*insrb*-MO	ACTGAAAGGACCACACTCACGCTTC
(exon7-intron7 junction)	
Control-MO	CCTCTTACCTCAGTTACAATTTATA
**Genotyping primer**	**Primer sequence**
SB-*insra*-MO	GAGCTCCACAACAAGTGCAA
	CTCCAGCTGTCCCAGGTTAG
SB-*insrb*-MO	ACACAAATCCGCACCATGAG
	CAATGGCCCAGCTGTTAGAG

Table S3. Morpholinos and the genotyping primers for zebrafish *insra/insrb* morpholinos.
